# Macrophages exert homeostatic actions in pregnancy to protect against preterm birth and fetal inflammatory injury

**DOI:** 10.1172/jci.insight.146089

**Published:** 2021-10-08

**Authors:** Nardhy Gomez-Lopez, Valeria Garcia-Flores, Peck Yin Chin, Holly M. Groome, Melanie T. Bijland, Kerrilyn R. Diener, Roberto Romero, Sarah A. Robertson

**Affiliations:** 1 Robinson Research Institute and Adelaide Medical School, The University of Adelaide, Adelaide, South Australia, Australia.; 2 Perinatology Research Branch, Division of Obstetrics and Maternal-Fetal Medicine, Division of Intramural Research, Eunice Kennedy Shriver National Institute of Child Health and Human Development, NIH, US Department of Health and Human Services; Bethesda, Maryland, and Detroit, Michigan, USA.; 3 Department of Obstetrics and Gynecology and; 4 Department of Biochemistry, Microbiology and Immunology, Wayne State University School of Medicine, Detroit, Michigan, USA.; 5 University of South Australia Cancer Research Institute, Clinical and Health Sciences, University of South Australia, Adelaide, South Australia, Australia.; 6 Department of Obstetrics and Gynecology, University of Michigan Medical School, Ann Arbor, Michigan, USA.; 7 Department of Epidemiology and Biostatistics, College of Human Medicine, Michigan State University, East Lansing, Michigan, USA.; 8 Center for Molecular Medicine and Genetics, Wayne State University School of Medicine, Detroit, Michigan, USA.; 9 Detroit Medical Center, Detroit, Michigan, USA.

**Keywords:** Reproductive Biology, Cytokines, Macrophages, Obstetrics/gynecology

## Abstract

Macrophages are commonly thought to contribute to the pathophysiology of preterm labor by amplifying inflammation — but a protective role has not previously been considered to our knowledge. We hypothesized that given their antiinflammatory capability in early pregnancy, macrophages exert essential roles in maintenance of late gestation and that insufficient macrophages may predispose individuals to spontaneous preterm labor and adverse neonatal outcomes. Here, we showed that women with spontaneous preterm birth had reduced CD209^+^CD206^+^ expression in alternatively activated CD45^+^CD14^+^ICAM3^–^ macrophages and increased TNF expression in proinflammatory CD45^+^CD14^+^CD80^+^HLA-DR^+^ macrophages in the uterine decidua at the materno-fetal interface. In *Cd11b^DTR/DTR^* mice, depletion of maternal CD11b^+^ myeloid cells caused preterm birth, neonatal death, and postnatal growth impairment, accompanied by uterine cytokine and leukocyte changes indicative of a proinflammatory response, while adoptive transfer of WT macrophages prevented preterm birth and partially rescued neonatal loss. In a model of intra-amniotic inflammation–induced preterm birth, macrophages polarized in vitro to an M2 phenotype showed superior capacity over nonpolarized macrophages to reduce uterine and fetal inflammation, prevent preterm birth, and improve neonatal survival. We conclude that macrophages exert a critical homeostatic regulatory role in late gestation and are implicated as a determinant of susceptibility to spontaneous preterm birth and fetal inflammatory injury.

## Graphical abstract

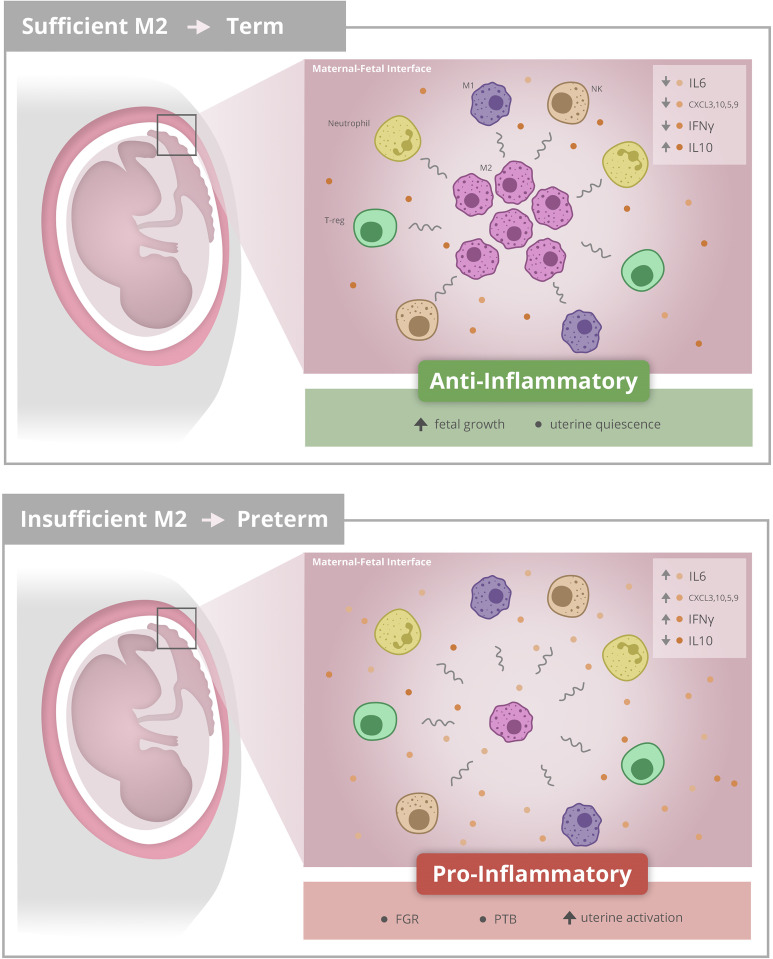

## Introduction

Preterm birth affects approximately 15 million infants annually and is the most common cause of death in children under 5 years of age (1, 2). Preterm neonates are at high risk of complications, including ongoing neurological and developmental disabilities (3, 4). The majority of preterm births are spontaneous (65%–70%), and the remainder (30%–35%) are iatrogenic (5–7). Spontaneous preterm births are preceded by preterm labor (5, 8, 9). The pathophysiological mechanisms that lead to preterm labor and the factors that confer susceptibility or resilience must be defined in order to identify effective strategies to prevent preterm birth.

Preterm labor is a syndrome of multiple etiologies (6), but a large subset of spontaneous preterm births is triggered by infectious or sterile insults that converge on immune and inflammatory mechanisms. Proinflammatory mediators convert the uterus from a quiescent to contractile state (10–13) and exert fetal inflammatory injury that exacerbates the neonatal consequences of prematurity (14, 15). In preterm labor, macrophages are conventionally thought to promote pathology by exerting proinflammatory functions that stimulate uterine contractility and cervical ripening (16–18). Macrophages with a proinflammatory phenotype are enriched in the uterine decidua of women who undergo spontaneous preterm labor (16, 19) and in the uterus and cervix of rodents administered proinflammatory stimuli to model preterm birth (20–22). Elevated proinflammatory cytokines, notably IL-6, IL-1β, and TNF, are detected prior to preterm birth in the uterus, placenta, amniotic fluid, and fetal tissues, and macrophages are implicated as a primary source and target population of these factors (20, 23–25). Macrophages also produce matrix metalloproteinases thought to assist in cervical ripening and rupture of the fetal membranes (26), and the kinetics of their recruitment are consistent with postpartum tissue remodeling and repair (22, 23). Nevertheless, animal experiments designed to investigate actions of proinflammatory macrophages in the progression of labor have not provided definitive evidence of a rate-limiting role for macrophages in labor initiation (27, 28).

Increasingly, macrophages are understood to exert not only immune-regulatory roles but also trophic and remodeling roles that facilitate development and maintain homeostasis in a wide range of peripheral tissues (29, 30). Earlier in pregnancy, uterine macrophage populations with alternatively activated, homeostatic phenotypes are involved in embryo implantation and placental development and defense against placental infection in mice (31–33) and humans (34–37).

We proposed that in late gestation, uterine macrophages may exert not only proinflammatory actions but also antiinflammatory homeostatic effects to sustain gestation prior to labor initiation at term. In the current study, we hypothesized that macrophages have essential roles in maintenance of late-gestation pregnancy, and that insufficient macrophages may predispose individuals to spontaneous preterm labor and adverse neonatal outcomes. To test this hypothesis, we undertook a series of experiments including 1) immunophenotyping of decidual macrophages from women with either term or spontaneous preterm birth, 2) targeted depletion of CD11b^+^ macrophages in *Cd11b^DTR/DTR^* transgenic mice, and 3) adoptive transfer of in vitro M2-polarized macrophages into mice administered intra-amniotic LPS to induce preterm birth. The results demonstrated a hitherto unappreciated homeostatic role for macrophages in late gestation, exerted by suppression of proinflammatory cytokines to maintain uterine quiescence, sustain pregnancy, and protect the fetus from premature birth and inflammatory injury that compromise normal development and postnatal survival.

## Results

### Altered macrophage phenotypes at the materno-fetal interface in women with spontaneous preterm birth.

**Figure 1:**
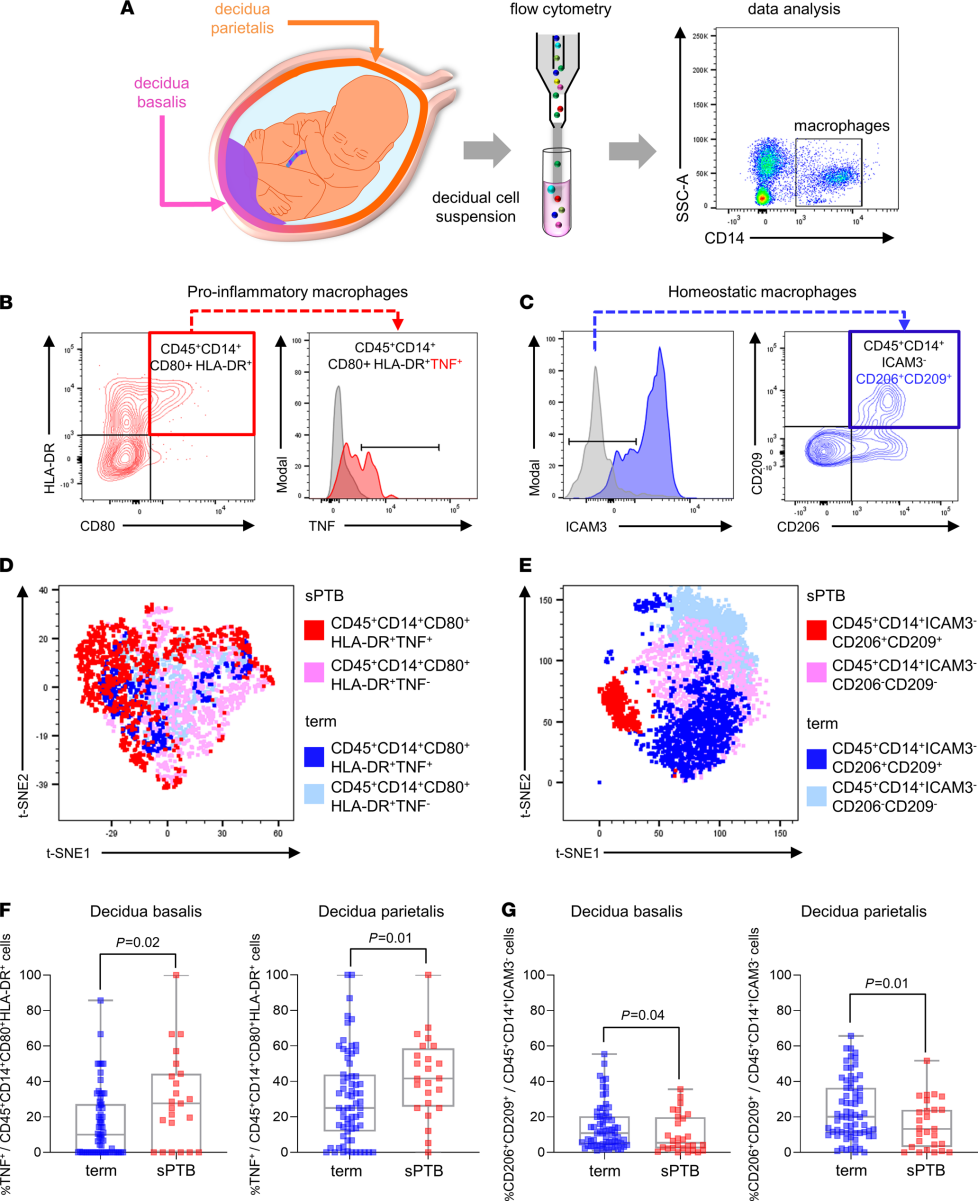
Specific subsets of proinflammatory and homeostatic macrophage subsets are differentially abundant at the materno-fetal interface of women with spontaneous preterm birth compared with term birth. (**A**) Schematic representation of the materno-fetal interface, including the uterine decidua basalis and decidua parietalis. Cell suspensions were prepared from the decidual tissues, and macrophage subsets were analyzed by flow cytometry with markers CD14, CD80, HLA-DR, ICAM3, CD206, CD209, CD163, NRP-1, TNF, IL12, iNOS, and IL10, according to the gating strategy in Supplemental Figure 1. (**B**) Representative FACS plots of CD45^+^CD14^+^CD80^+^HLA-DR^+^ cells, considered proinflammatory macrophages, expressing TNF. (**C**) Representative FACS plots of CD45^+^CD14^+^ICAM3^–^ cells, considered homeostatic macrophages, expressing CD206^+^CD209^+^. (**D**) Representative t-distributed stochastic neighbor embedding (t-SNE) plot of CD45^+^CD14^+^CD80^+^HLA-DR^+^ proinflammatory macrophages, color-coded to identify TNF^+^ cells from women with spontaneous preterm birth (red) and term birth (dark blue), and TNF^–^ cells from women with spontaneous preterm birth (pink) and term birth (light blue). (**E**) Representative t-SNE plot of CD45^+^CD14^+^ICAM^–^ homeostatic macrophages, color-coded to identify CD206^+^CD209^+^ cells from women with spontaneous preterm birth (red) and term birth (dark blue), and CD206^–^CD209^–^ cells from women with spontaneous preterm birth (pink) and term birth (light blue). (**F**) Frequency of CD45^+^CD14^+^CD80^+^HLA-DR^+^ cells expressing TNF in the decidua basalis and decidua parietalis of women with term (*n* = 63–67) or spontaneous preterm (*n* = 23–24) birth. (**G**) Frequency of CD45^+^CD14^+^ICAM3^–^ cells expressing CD206 and CD209 in the decidua basalis or the decidua parietalis of women with term (*n* = 66–68) or spontaneous preterm (*n* = 27–28) birth. Data are presented as mean and SEM. Symbols are values from individual women. Data were analyzed by Mann-Whitney *U* test. *P* values were considered significant when *P* was less than 0.05. Data on other macrophage subpopulations are shown in Supplemental Figure 2. Demographic and clinical characteristics of the study population are shown in Table 1.

Decidual macrophages with homeostatic properties are present in the uterus in early (35) and late (19) pregnancy and have the potential to contribute to the immune environment that sustains pregnancy tolerance to on-time birth (19, 35–38). We used flow cytometry to investigate the phenotypes of macrophages in uterine decidual tissues (Figure 1A) at the time of delivery in *n* = 69 women with a term birth (mean gestational age at delivery = 39.1 weeks) and *n* = 29 women with a spontaneous preterm birth (mean gestational age at delivery = 35.1 weeks). Women were matched for age, BMI, race, and delivery mode (Table 1). Most women included in this study were African American, a high-risk population for preterm birth (39).

Several macrophage subpopulations were identified in both the decidua basalis and the decidua parietalis (Supplemental Figure 1, A and B; supplemental material available online with this article; https://doi.org/10.1172/jci.insight.146089DS1). Macrophages with a proinflammatory phenotype (CD45^+^CD14^+^CD80^+^HLA-DR^+^ cells; refs. 19, 40, 41) and macrophages with an alternatively activated/homeostatic phenotype (CD45^+^CD14^+^ICAM3^–^ cells; ref. 35) (Figure 1, B and C) both exhibited different phenotypic features in women with spontaneous preterm birth than in those who delivered at term (Figure 1, D and E). Notably, macrophages with a proinflammatory phenotype more commonly expressed TNF in both the decidua basalis and the decidua parietalis of women with spontaneous preterm birth (Figure 1F). Among macrophages expressing the CD45^+^CD14^+^ICAM3^–^ alternatively activated phenotype, fewer expressed activation markers CD206^+^CD209^+^, again in both tissue compartments (Figure 1G). No changes were apparent in the relative proportions of the parent CD45^+^CD14^+^CD80^+^HLA-DR^+^ and CD45^+^CD14^+^ICAM3^–^ macrophage populations (Supplemental Figure 2, A and B); or proinflammatory macrophages expressing iNOS or IL-12; or homeostatic macrophages expressing CD163, IL-10, or NRP-1 (Supplemental Figure 2, C and D). Furthermore, in a third group of women with preterm birth due to iatrogenic causes (*n* = 11, Supplemental Table 1), analysis of decidual macrophages showed no change in TNF expression in CD45^+^CD14^+^CD80^+^HLA-DR^+^ cells (Supplemental Figure 3A). Although the frequency of CD45^+^CD14^+^ICAM3^–^CD206^+^CD209^+^ cells was reduced in the decidua basalis compared with term tissues (Supplemental Figure 3B), this change was not evident in the larger decidua parietalis compartment. These findings indicate a specific change in the activation status of decidual macrophages in women who underwent spontaneous preterm birth, with a shift away from a homeostatic and toward a proinflammatory state. This finding prompted us to explore the physiological requirement for macrophages in late gestation using an in vivo model.

### CD11b^+^ macrophage depletion in late gestation causes preterm birth and adverse neonatal outcomes.

**Figure 2:**
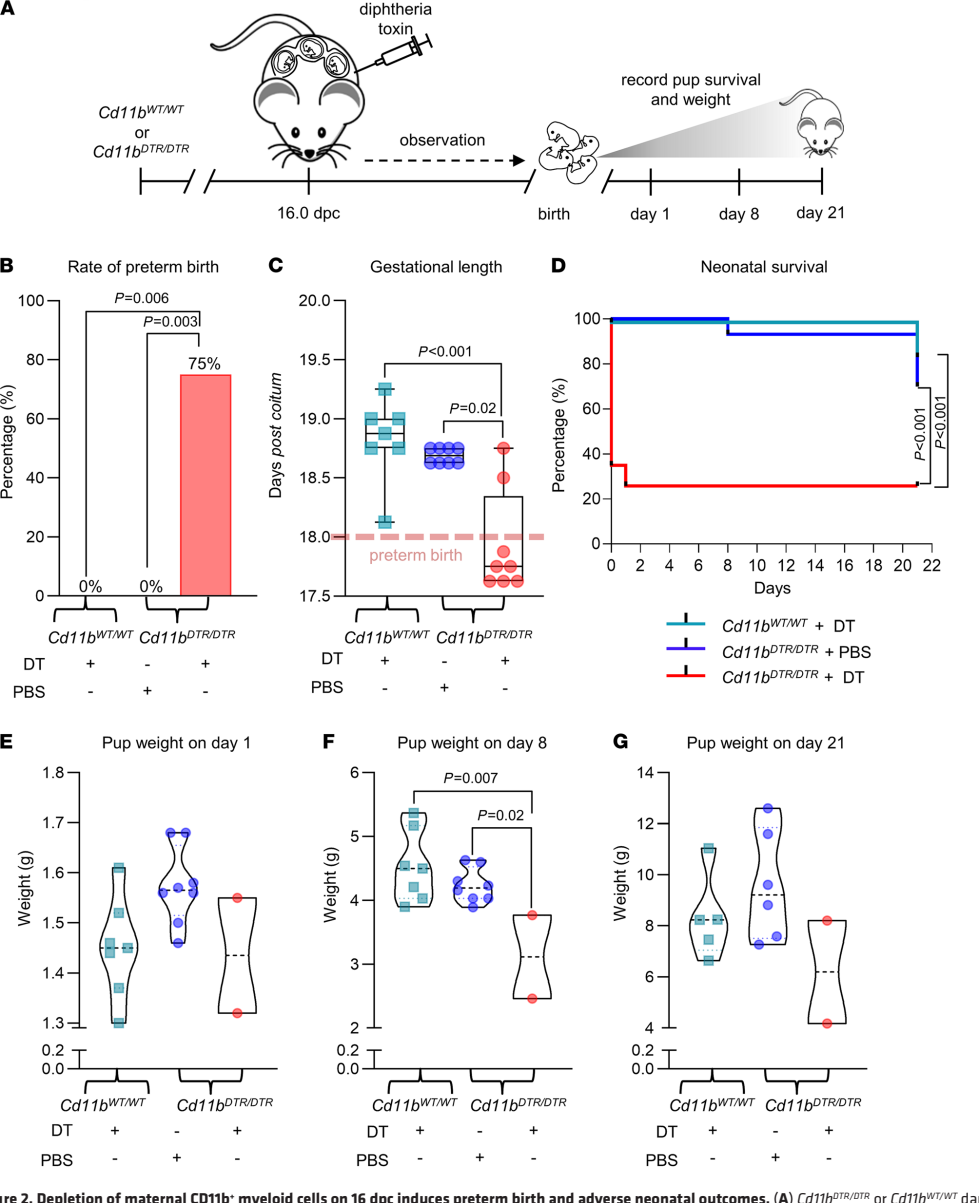
Depletion of maternal CD11b^+^ myeloid cells on 16 dpc induces preterm birth and adverse neonatal outcomes. (**A**) *Cd11b^DTR/DTR^* or *Cd11b^WT/WT^* dams were administered diphtheria toxin (DT, 25 ng/g, i.p.) or PBS on 16 dpc. Birth and neonatal outcomes were recorded (all *n* = 7–8 dams per group). Parameters shown are (**B**) rate of preterm birth (delivery within 48 hours of intervention, e.g., ≤18 dpc), analyzed by Fisher’s exact test; (**C**) gestational length presented as box plots where midlines indicate medians, boxes indicate IQR, and whiskers indicate minimum/maximum range, analyzed by Kruskal-Wallis test followed by Mann-Whitney *U* test; and (**D**) Kaplan-Meier survival curves showing the percentage survival per litter of neonates at 1, 8, and 21 days postpartum, analyzed by Mantel-Cox tests. (**E**–**G**) Violin plots showing the mean weight per litter of surviving pups at 1, 8, and 21 days postpartum. Symbols are median values from individual dams. Data were analyzed by 1-way ANOVA and post hoc 2-tailed *t* test. *P* values were considered significant when *P* was less than 0.05.

To investigate the effects of macrophage depletion in late gestation, we used *Cd11b^DTR/DTR^* transgenic mice to allow acute, transient depletion of CD11b^+^ macrophages upon administration of diphtheria toxin (DT) (42, 43). *Cd11b^DTR/DTR^* mice and control WT *Cd11b^WT/WT^* mice were mated with BALB/c males to achieve allogeneic pregnancy, and then administered DT or PBS (vehicle control) on 16 dpc (3 days prior to expected birth, approximately equivalent to 34 weeks’ gestation in humans), and pregnancy and neonatal outcomes were assessed (Figure 2A). Administration of DT on 16 dpc resulted in preterm birth (delivery of pups within 48 hours of DT administration) in 75% of *CD11b^DTR/DTR^* dams but did not affect timing of birth in *CD11b*^WT/WT^ dams (Figure 2, B and C), consistent with a mechanism involving CD11b^+^ cell depletion. Control *CD11b*^WT/WT^ dams given DT and *Cd11b^DTR/DTR^* dams given PBS all delivered on time at term (Figure 2C).

The viability and health of pups born to *CD11b^DTR/DTR^* mice given DT were severely affected, but pups of *CD11b*^WT/WT^ mice given DT were healthy. *CD11b^DTR/DTR^* and *CD11b*^WT/WT^ dams given DT and *CD11b^DTR/DTR^* dams given PBS all delivered a similar number of pups (data not shown), but in *CD11b^DTR/DTR^* mice given DT, 74% of pups were either born dead or failed to survive the first 24 hours compared with control groups, for which postnatal survival was 100% (Figure 2D). Perinatal loss is a common feature of mouse preterm birth models (44, 45) and is likely attributable to the developmental immaturity of pups born before 18 dpc, as well as exposure to maternal inflammatory mediators that cause placental and/or fetal inflammatory injury (15). Surviving pups from 2 *CD11b^DTR/DTR^* mice given DT that delivered after 18 dpc showed impaired growth in the early postnatal phase compared with controls (Figure 2, E and F), although this was partially normalized by 21 days of age (Figure 2G).

Maternal DT administration later in gestation (at 17 dpc) also shortened gestational length (Supplemental Figure 4A) and decreased neonatal survival (Supplemental Figure 4B), but not to the same extent as depletion at 16 dpc, potentially because fetuses were more developmentally mature at this time. Moreover, DT administration on 17 dpc caused reduced birth weight followed by catch-up growth in the postnatal phase (Supplemental Figure 4, C–E). These data suggest that CD11b^+^ macrophages may be essential for sustaining late gestation and ensuring optimal fetal development, neonatal survival, and a normal postnatal growth trajectory.

### Maternal macrophages, but not fetal macrophages, are implicated in preterm birth.

**Figure 3:**
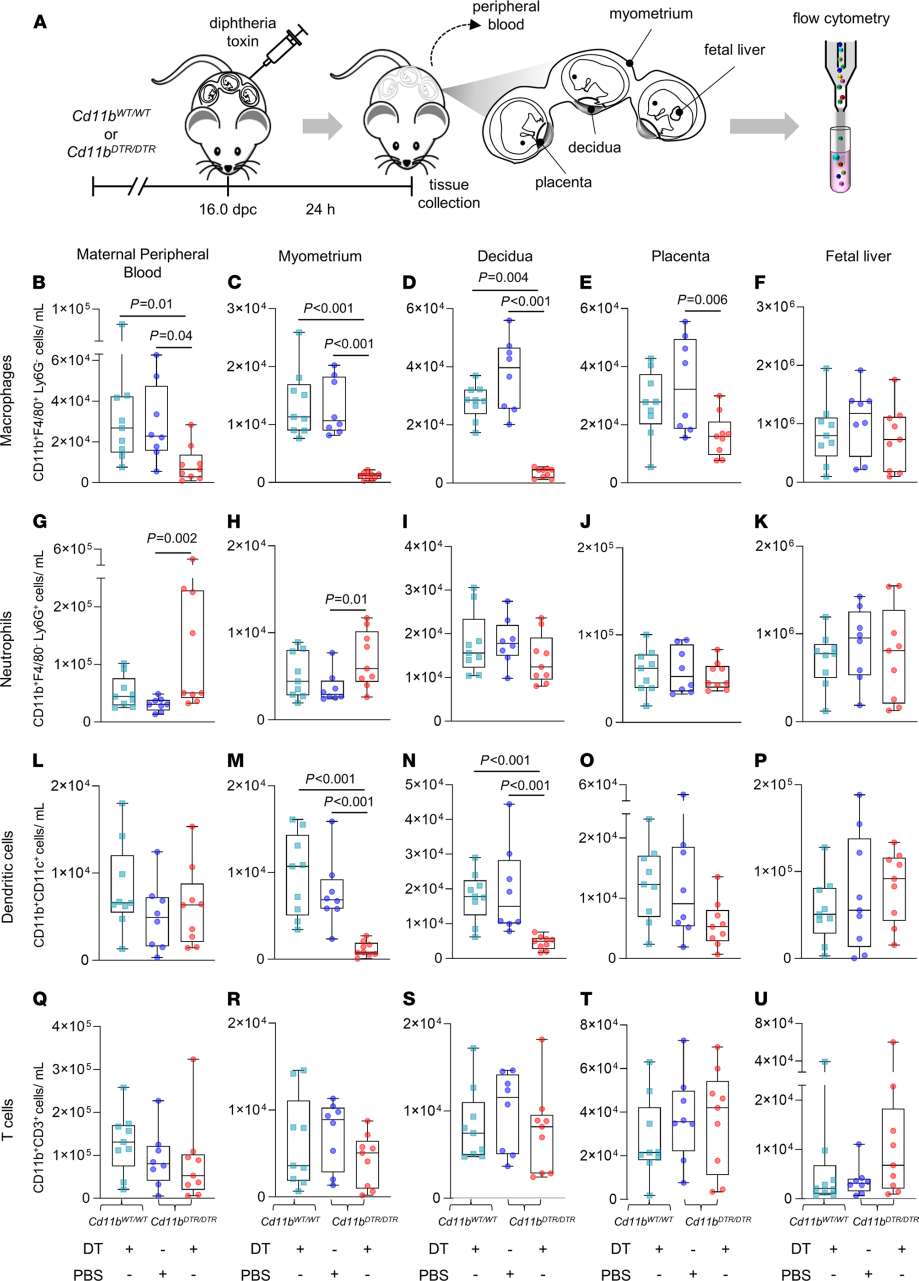
Effects of depletion of maternal CD11b^+^ myeloid cells on leukocyte populations in maternal and fetal tissues. (**A**) *Cd11b^DTR/DTR^* or *Cd11b^WT/WT^* dams were administered diphtheria toxin (DT, 25 ng/g, i.p.) or PBS on 16 dpc. Tissues were collected 24 hours later for analysis of leukocytes by flow cytometry with markers CD45, CD11b, F4/80, Ly6G, CD11c, CD3, CD4, and CD8, according to the gating strategy in Supplemental Figure 6, to quantify (**B**–**F**) macrophages, (**G**–**K**) neutrophils, (**L**–**P**) DCs, and (**Q**–**U**) T cells in the maternal peripheral blood, uterine myometrium, uterine decidua, placenta, and fetal liver (*n* = 8–9 per group). Data are presented as box plots where the midline indicates the median, the box indicates IQR, and whiskers indicate the minimum/maximum range. Symbols are values from individual dams. Data were analyzed by 1-way ANOVA and post hoc 2-tailed *t* test. *P* values were considered significant when *P* was less than 0.05. Data on macrophages, neutrophils, DCs, and T cells as a proportion of total CD45^+^ cells are shown in Supplemental Figure 6. Data on CD4^+^ and CD8^+^ T cell subsets are shown in Supplemental Figure 8.

Next, we investigated the specific lineages of uterine and placental cells affected by DT administration in *Cd11b^DTR/DTR^* dams (Figure 3A). Immunohistochemical analysis showed that 24 hours after DT administration, more than 95% of cells expressing the macrophage lineage marker F4/80 were depleted from implantation sites of *Cd11b^DTR/DTR^* dams (Supplemental Figure 5). Within the placental labyrinth tissue, F4/80^+^ cells in the intervillous maternal blood space were absent from *Cd11b^DTR/DTR^* dams given DT, while the majority of F4/80^+^ cells located in the interstitial villous tissue, presumed to be fetal Hofbauer cells, were retained (Supplemental Figure 5). Flow cytometry analysis (Supplemental Figure 6A) showed that the number and proportion of macrophages (CD11b^+^F4/80^+^ cells) were reduced by more than 90% in the maternal peripheral blood, myometrium, and decidua 24 hours after DT administration in *Cd11b^DTR/DTR^* dams compared with control *CD11b^DTR/DTR^* dams given PBS or *CD11b*^WT/WT^ dams given DT (Figure 3, B–D; and Supplemental Figure 6, B–D). Total placental macrophages were reduced by approximately 30% in cell number, whereas fetal liver macrophages were unchanged in *Cd11b^DTR/DTR^* dams given DT (Figure 3, E and F; and Supplemental Figure 6, E and F). These data are consistent with loss of *Cd11b^DTR/DTR^* maternal macrophages and retention of *Cd11b^DTR/+^* fetally derived macrophages in the placenta and fetal liver.

A limitation of the *CD11b^DTR/DTR^* model is that CD11b^+^ cells other than macrophages can be affected after DT administration because CD11b is expressed by some neutrophils and DCs (46). Neutrophils (CD11b^+^Ly6G^+^ cells) were elevated to a variable degree in the maternal peripheral blood and myometrium of some *Cd11b^DTR/DTR^* dams after DT administration, but neutrophil recruitment was not evident in the decidua (Figure 3, G–I; and Supplemental Figure 6, G–I). In the placenta and fetal liver, neutrophils were not affected (Figure 3, J and K; and Supplemental Figure 6, J and K). CD11b^+^CD11c^+^ cells in the myometrium and decidua were reduced by 60%–80% after DT administration in *Cd11b^DTR/DTR^* dams compared with control mice (Figure 3, M and N; and Supplemental Figure 6, M and N), and a reduction was also seen in maternal blood when considered as a proportion of total CD45^+^ leukocytes (Figure 3L and Supplemental Figure 6L). CD11b^+^CD11c^+^ myeloid DCs were moderately depleted from the placenta when considered as a proportion of CD45^+^ leukocytes, but not when absolute placental DC numbers were quantified (Figure 3O and Supplemental Figure 6O). Myeloid DCs were not changed in the fetal liver (Figure 3P and Supplemental Figure 6P).

Uterine F4/80^+^ macrophages and myeloid DCs exhibit a close ontogenic relationship and share phenotypic features (47), such that many uterine macrophages express MHC class II and have the potential to modulate T cell activation and phenotype (48). Many F4/80^+^ macrophages recovered from the myometrium, decidua, and placenta on day 17 dpc also expressed MHC class II (Supplemental Figure 7), indicating potential to modulate T cells in late gestation. However, T cell numbers were not changed in the maternal blood, myometrium, or decidua (Figure 3, Q–S; and Supplemental Figure 6, Q–S), or in the placenta or fetal liver (Figure 3, T and U; and Supplemental Figure 6, T and U), after DT administration. Likewise, no changes were observed in the number or proportion of CD4^+^ and CD8^+^ T cell subsets upon DT administration (Supplemental Figure 8, A–U).

Together, these findings confirmed that the acute systemic depletion of CD11b^+^ cells primarily affected macrophages and myeloid DCs in the maternal compartment. This ablation of CD11b^+^ myeloid cells was accompanied by functional shifts in neutrophils in the myometrium and the maternal circulation, but not in decidual tissue. Importantly, administration of DT to dams did not substantially alter fetal leukocytes. These data support the interpretation that loss of maternal CD11b^+^ myeloid cells is primarily responsible for preterm birth and adverse perinatal outcomes.

### Adoptive transfer of macrophages rescues preterm birth and improves neonatal survival.

**Figure 4:**
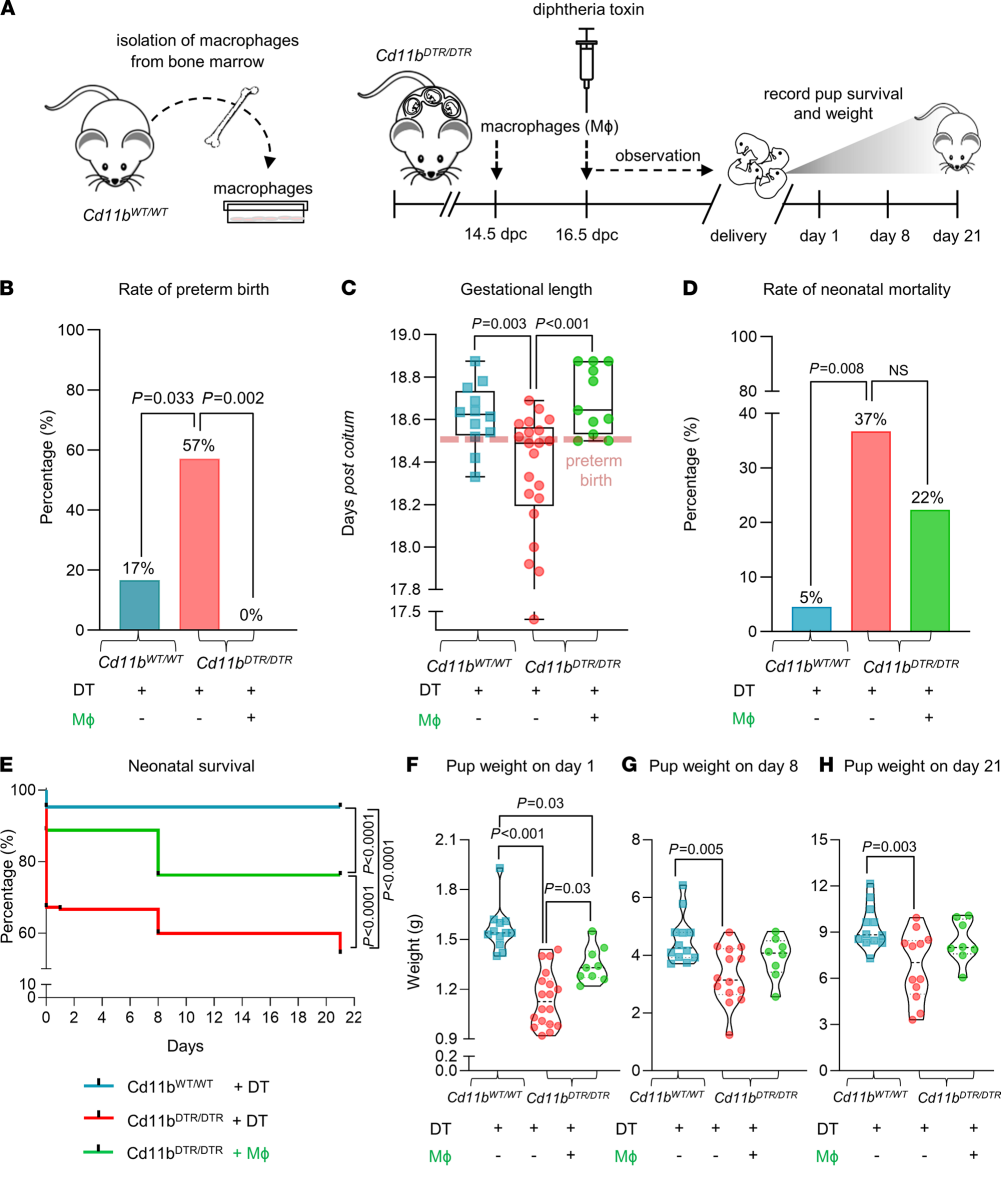
Adoptive transfer of macrophages prevents preterm birth but does not restore adverse neonatal outcomes after depletion of maternal CD11b^+^ myeloid cells. (**A**) Bone marrow cells were collected from *Cd11b^WT/WT^* mice and differentiated into nonpolarized macrophages (Mϕ) by in vitro culture in complete RPMI medium. Nonpolarized macrophages or vehicle were adoptively i.v. transferred to *Cd11b^DTR/DTR^* dams on 14.5 dpc and 16.5 dpc, followed by diphtheria toxin administration (DT, 25 ng/g, i.p.) on 16.5 dpc. Control *Cd11b^WT/WT^* females were administered vehicle and DT (25 ng/g, i.p.). Birth and neonatal outcomes were recorded (all *n* = 11–21 dams per group). Parameters shown are (**B**) rate of preterm birth (delivery within 48 hours of intervention, e.g., ≤18.5 dpc), analyzed by Fisher’s exact test; (**C**) gestational lengths presented as box plots where midlines indicate medians, boxes indicate IQR, and whiskers indicate minimum/maximum range, analyzed by 1-way ANOVA and post hoc 2-tailed *t* test; (**D**) neonatal mortality per litter of pups, analyzed by Mann-Whitney *U* tests; and (**E**) Kaplan-Meier survival curves showing the percentage of survival per litter from day 1 until day 21 after birth of neonates, analyzed by Mantel-Cox test. (**F**–**H**) Violin plots (medians) showing the mean weight per litter of neonates at 1, 8, and 21 days postpartum. Symbols are median values from individual dams. Data were analyzed by 1-way ANOVA and post hoc 2-tailed *t* test.

To specifically address whether macrophages are the key lineage responsible for preterm birth after depletion of CD11b^+^ myeloid cells from *Cd11b^DTR/DTR^* dams, we evaluated whether adoptive transfer of WT macrophages could restore the timing of parturition and prevent neonatal mortality and morbidity. WT bone marrow–derived macrophages (BMDMs) were generated in vitro and adoptively transferred to DT-injected *Cd11b^DTR/DTR^* dams (Figure 4A). As previously observed, CD11b^+^ myeloid cell depletion reduced the gestational length and induced preterm birth (Figure 4, B and C), albeit with slightly later timing due to DT administration occurring approximately 12 hours later to accommodate technical considerations. Adoptive transfer of macrophages protected against preterm birth and restored gestational length, causing all dams to deliver at term (Figure 4, B and C). Additionally, perinatal loss was partially restored by adoptive transfer of macrophages (Figure 4, D and E), but postnatal birth weight and growth impairment caused by CD11b^+^ myeloid cell depletion were only partially improved (Figure 4, F–H). These findings showed that maternal macrophage loss was responsible for the adverse perinatal outcomes elicited by CD11b^+^ myeloid cell depletion in *Cd11b^DTR/DTR^* dams, as opposed to myeloid DC loss or off-target effects of DT treatment. However, the postnatal effects of maternal CD11b^+^ myeloid cell depletion were not fully reversed upon macrophage restoration. Thus, we investigated the mechanisms by which CD11b^+^ myeloid cell depletion caused preterm birth, then evaluated in vitro strategies to boost the potency of macrophages to improve pregnancy and neonatal outcomes.

### Proinflammatory mediators and leukocyte activation are implicated in preterm birth.

**Figure 5:**
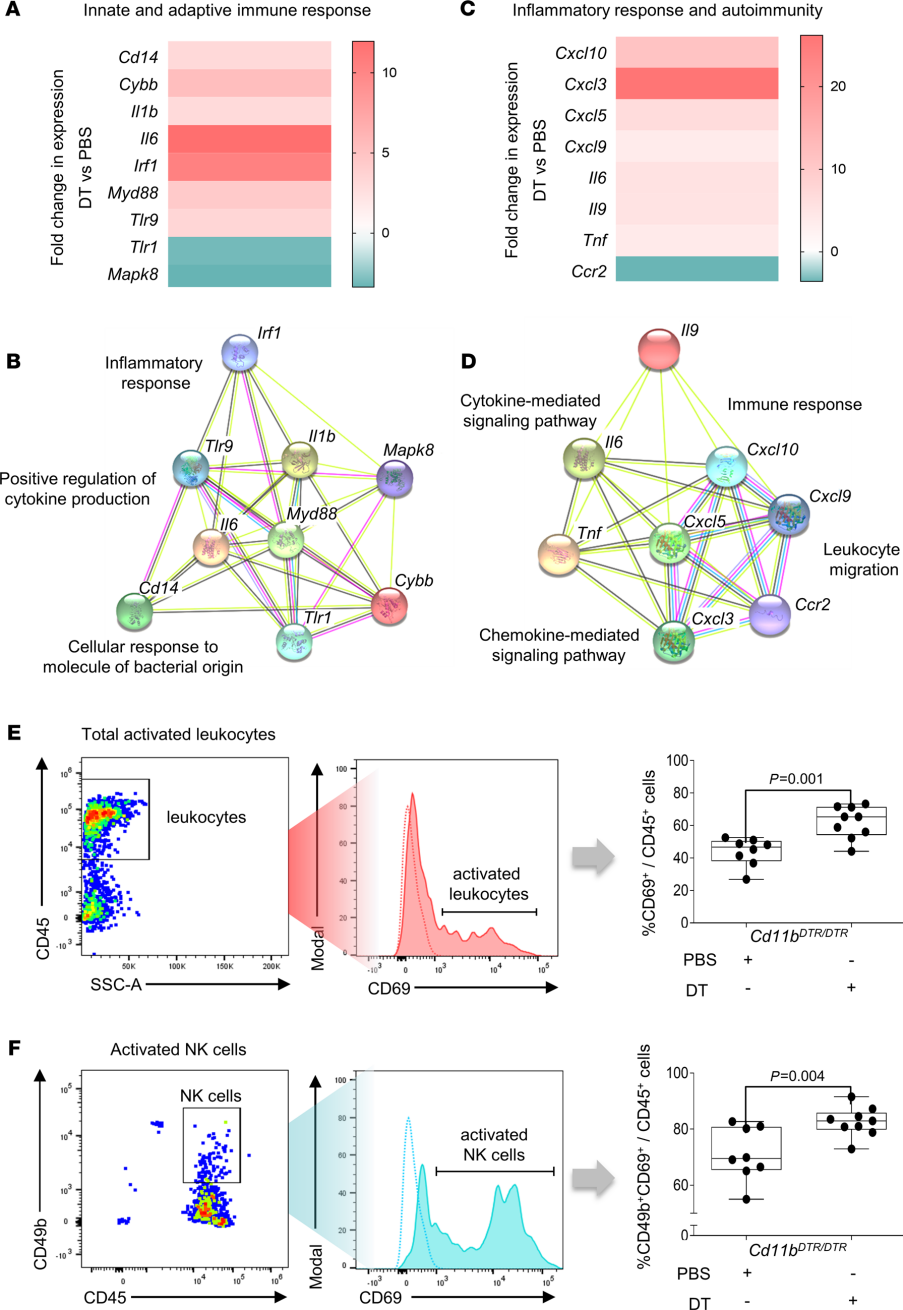
Depletion of maternal CD11b^+^ myeloid cells promotes upregulation of inflammatory mediators and leukocyte activation at the materno-fetal interface. *Cd11b^DTR/DTR^* dams were administered diphtheria toxin (DT, 25 ng/g, i.p.) or PBS on 16 dpc. The uterine decidua was collected 24 hours later for gene expression profiling using RT-qPCR arrays (tissue pooled from *n* = 7–8 per group) and for analysis of leukocytes by flow cytometry (*n* = 7–8 per group). (**A**) Heatmap of differentially expressed genes and (**B**) interaction network obtained by STRING analysis of pathways attenuated in the decidua after depletion of CD11b^+^ cells, obtained using the “innate and adaptive immune response” array. (**C**) Heatmap of differentially expressed genes and (**D**) interaction network obtained by STRING analysis of pathways attenuated in the decidua after depletion of CD11b^+^ cells, obtained using the “inflammatory response and autoimmunity” array. Flow cytometry gating strategy for determining the effect of CD11b^+^ cell depletion on proportions of (**E**) activated leukocytes (CD69^+^/CD45^+^ cells) and (**F**) NK cells (CD49b^+^CD69^+^/CD45^+^ cells) in decidual tissues. Data are presented as box plots where the midline indicates the median, the box indicates the IQR, and whiskers indicate the minimum/maximum range. Symbols are values from individual dams. Data were analyzed by Mann-Whitney *U* tests. *P* values were considered significant when *P* was less than 0.05.

To investigate the specific molecular and cellular changes induced by CD11b^+^ myeloid cell depletion, decidual tissues were collected from *Cd11b^DTR/DTR^* dams 24 hours after DT administration. In an exploratory analysis, changes in expression of proinflammatory mediators were evaluated in pooled tissue samples using 2 specific quantitative reverse transcription PCR (RT-qPCR) arrays. The first array, targeting innate and adaptive immune response genes, revealed extensive (more than 8–10-fold) upregulation of *Il6* and *Irf1*, as well as elevated *Cd14*, *Tlr9*, and *Il1b* upon CD11b^+^ myeloid cell depletion (Figure 5A). STRING analysis of these upregulated mediators showed enrichment of networks, including inflammatory response, positive regulation of cytokine production, and cellular response to molecule of bacterial origin (Figure 5B). The second array, targeting inflammatory response and autoimmunity genes, showed more than 20-fold induction of *Cxcl3*, as well as increased *Cxcl5*, *Cxcl9*, *Cxcl10*, *Tnf*, and *Il9*, upon CD11b^+^ myeloid cell depletion (Figure 5C). Enriched STRING networks for these genes were immune response, cytokine-mediated signaling pathway, leukocyte migration, and chemokine-mediated signaling pathway (Figure 5D).

We next evaluated expression of the activation marker CD69 in decidual leukocytes to determine the impact of maternal CD11b^+^ myeloid cell depletion on decidual leukocyte activation. CD69 was upregulated on decidual leukocytes in CD11b^+^ myeloid cell–depleted dams (Figure 5E), with elevated activation of NK cells (Figure 5F). These analyses indicate that CD11b^+^ myeloid cell depletion resulted in upregulation of inflammatory mediators as well as activation of NK cells at the materno-fetal interface, which are common features of the immune changes preceding preterm birth and the accompanying adverse neonatal outcomes (24, 27, 49).

### Progesterone rescues preterm birth but not adverse postnatal outcomes.

Progesterone has potent antiinflammatory effects (11, 50, 51), and premature demise of progesterone signaling can cause preterm birth (23). Previously, we showed that CD11b^+^ myeloid cell depletion from *CD11b^DTR/DTR^* mice in early pregnancy ablates ovarian synthesis of progesterone within 24 hours of DT administration (32), and others have shown that immune activation in early gestation elicits pregnancy loss through suppressing progesterone synthesis (52). To determine whether CD11b^+^ myeloid cell depletion in late gestation might act via progesterone, we measured plasma progesterone concentrations in DT-injected and control *Cd11b^DTR/DTR^* dams. No effect of DT administration at 16 dpc was seen on progesterone levels measured 24 hours later (Supplemental Figure 9A), and the precipitous decline in plasma progesterone, expected after 18 dpc in WT mice (53), did not occur prematurely after CD11b^+^ myeloid cell depletion. Nevertheless, given that progesterone can mitigate LPS-induced preterm birth in mice (54) and is an effective clinical intervention for some women at risk of preterm birth (55), we tested whether exogenous progesterone might alleviate the adverse pregnancy outcomes in CD11b^+^ myeloid cell–depleted dams (Supplemental Figure 9B). Progesterone protected *Cd11b^DTR/DTR^* dams from preterm birth caused by CD11b^+^ myeloid cell depletion and extended gestational length (Supplemental Figure 9, C and D). However, progesterone did not improve the survival of neonates born to CD11b^+^ myeloid cell–depleted dams (Supplemental Figure 9E) or attenuate growth impairment in surviving neonates (Supplemental Figure 9, F–H). These results showed that although progesterone supplementation can help sustain uterine quiescence caused by depletion of maternal CD11b^+^ myeloid cells, it is not adequate to overcome the adverse neonatal effects, implying a mechanism of macrophage action that is independent of progesterone.

### Adoptive transfer of in vitro M2-polarized macrophages prevents intra-amniotic inflammation–induced preterm birth and reduces neonatal mortality.

The findings in the *Cd11b^DTR/DTR^* model implicated macrophages as exerting an antiinflammatory/homeostatic role in order to sustain uterine quiescence. This is consistent with the finding that decidual macrophages displaying markers of alternative activation (i.e., an M2 phenotype) were enriched in women who delivered a healthy term neonate (Figure 1G). Therefore, we investigated whether in mice, macrophages at the materno-fetal interface also exhibit an antiinflammatory profile during late gestation. Macrophages were recovered from the uterine decidua and myometrium by cell sorting from WT mice, and their cytokine synthesis was evaluated (Supplemental Figure 10A). Decidual macrophages released high levels of antiinflammatory cytokines TGF-β and IL-10, the latter increasing as gestation progressed (Supplemental Figure 10B), accompanied by reduced proinflammatory IL-12 between 17.5 dpc and term (Supplemental Figure 10C). Myometrial macrophages displayed a similar cytokine profile (Supplemental Figure 10, D and E). Since IL-10 and IL-12 are antagonistically opposing and an elevated IL-10 to IL-12 ratio is an indicator of homeostatic M2 function (56, 57), this implies that decidual macrophages have antiinflammatory functions during late gestation.

**Figure 6:**
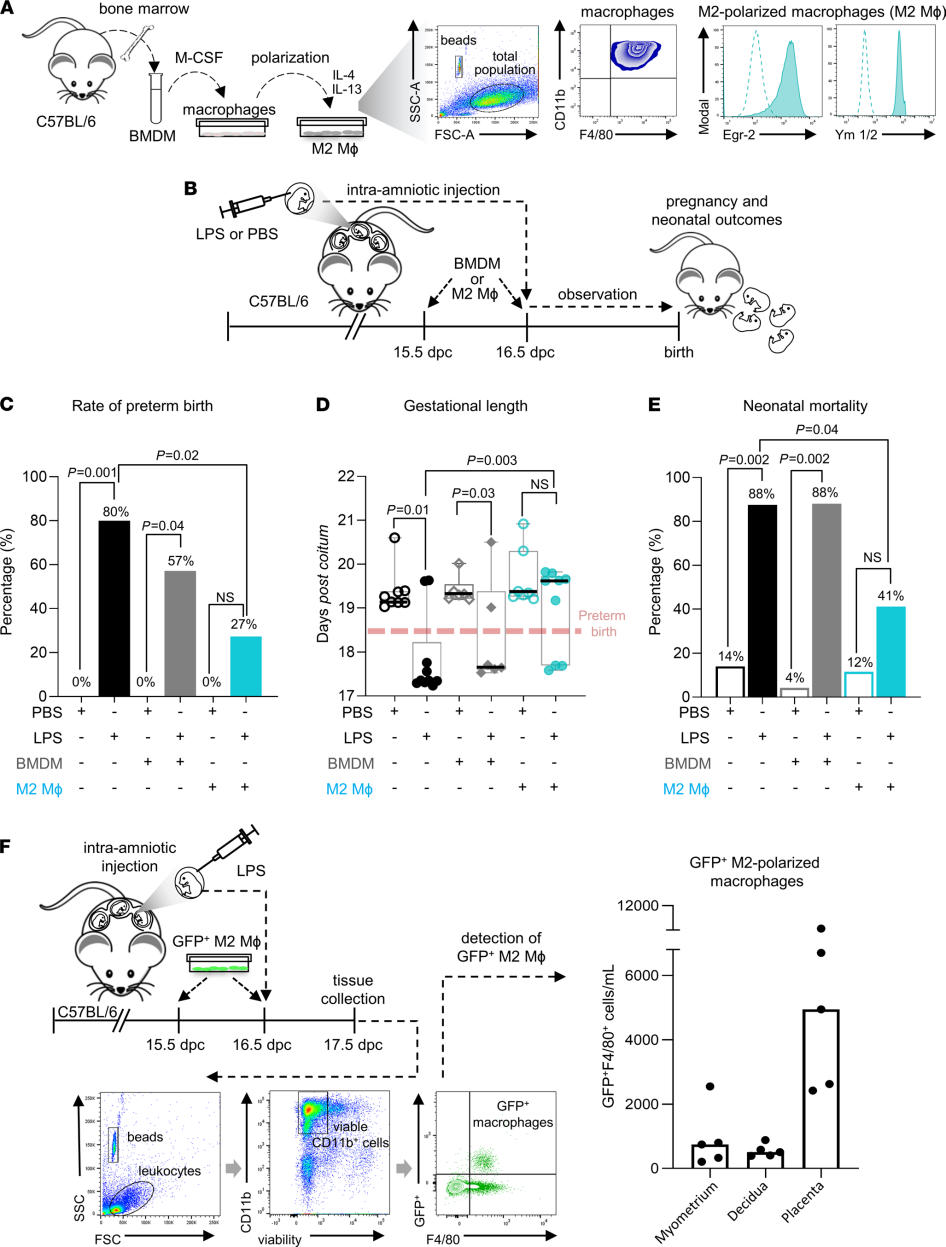
Adoptive transfer of M2-polarized macrophages prevents intra-amniotic inflammation–induced preterm birth and adverse neonatal outcomes. (**A**) Bone marrow–derived cells collected from C57BL/6 mice were differentiated into macrophages by in vitro culture with M-CSF followed by stimulation with IL-4 and IL-13 to induce M2 polarization. M2 polarization was confirmed by macrophage expression of Egr-2 and Ym1/2 analyzed by flow cytometry. Unpolarized bone marrow–derived macrophages (BMDMs) were also utilized. (**B**) M2-polarized macrophages (M2 Mϕ), BMDMs, or vehicle were i.v. administered on 15.5 dpc and 16.5 dpc to C57BL/6 dams followed by intra-amniotic injection with LPS or PBS on 16.5 dpc. Pregnancy and neonatal outcomes were recorded (*n* = 6–10 dams per group). Parameters shown are (**C**) rate of preterm birth (delivery within 48 hours of intervention, e.g., <18.5 dpc), analyzed by 1-tailed Fisher’s exact tests; (**D**) gestational length, presented as box plots where midlines indicate medians, boxes indicate IQR, and whiskers indicate minimum/maximum range, analyzed by Mann-Whitney *U* tests, and (**E**) neonatal mortality of pups per litter, analyzed by Mann-Whitney *U* tests. (**F**) Study design to detect GFP^+^ M2-polarized macrophages in maternal and fetal tissues. Bar plots representing the median numbers of adoptively transferred GFP^+^ M2-polarized macrophages in the myometrium, decidua, and placenta of recipient dams after intra-amniotic injection with LPS (*n* = 5). Symbols are values from individual dams. *P* values were considered significant when *P* was less than 0.05.

Next, we investigated whether macrophages polarized in vitro toward an M2 phenotype could protect against inflammation-induced preterm birth and adverse neonatal outcomes. We utilized a model of intra-amniotic inflammation and reasoned that treatment of macrophages to induce polarization toward an M2 antiinflammatory phenotype would maximize their effectiveness after transfer. The ability of IL-4 plus IL-13 to differentiate and polarize BMDMs to M2 macrophages in vitro was confirmed by their expression of the markers Egr-2 (58) and Ym1/2 (59) (Figure 6A). BMDMs without polarization were included to investigate the relative efficacy of M2-polarized versus nonpolarized macrophages. Macrophages were adoptively transferred to dams that received an ultrasound-guided intra-amniotic injection of LPS, an intervention that resembles the clinical scenario of intra-amniotic inflammation (45, 60, 61) (Figure 6B). Intra-amniotic inflammation caused preterm birth in 80% of dams. This rate decreased to 27% after adoptive transfer of M2-polarized macrophages but was not improved by nonpolarized BMDMs (Figure 6C). The reduced gestational length (Figure 6D) and elevated neonatal mortality (Figure 6E) elicited by LPS were also restored by transfer of M2-polarized macrophages but not BMDMs. Using GFP^+^ transgenic mice, we showed that transferred macrophages infiltrated the myometrial, decidual, and placental tissues (Figure 6F), although the number of cells recovered was at least an order of magnitude lower than seen in WT control mice, indicating that only partial macrophage replacement was achieved. Nevertheless, transfer of macrophages polarized in vitro toward an antiinflammatory M2 phenotype was surprisingly effective in protecting pregnancy and neonatal outcomes, to a substantially greater extent than was achieved with nonpolarized macrophages.

### Adoptive transfer of in vitro M2-polarized macrophages reduces maternal and intra-amniotic inflammatory mediators as well as fetal tissue inflammation.

**Figure 7:**
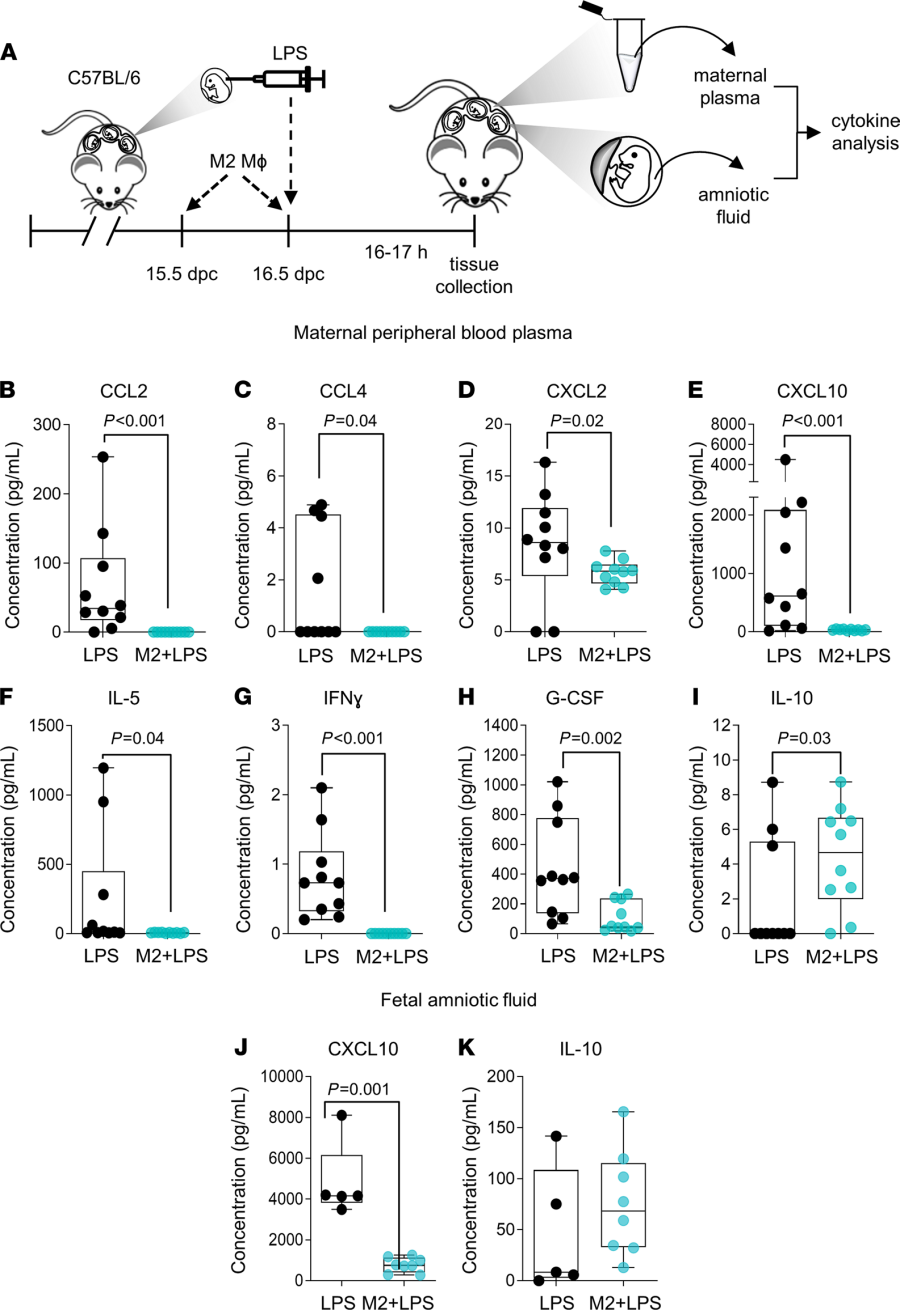
Adoptive transfer of M2-polarized macrophages to dams reduces inflammatory cytokines in maternal peripheral blood and fetal amniotic fluid. (**A**) M2-polarized macrophages (M2 Mɸ) or vehicle were i.v. administered on 15.5 dpc and 16.5 dpc to C57BL/6 dams followed by intra-amniotic injection with LPS on 16.5 dpc (M2+LPS). Control C57BL/6 dams were injected intra-amniotically with LPS only (LPS). Dams were euthanized 16 hours after LPS injection to collect maternal plasma and fetal amniotic fluid. Multiplex bead assays were used to determine the concentrations of CCL2 (**B**), CCL4 (**C**), CXCL2 (**D**), CXCL10 (**E**), IL-5 (**F**), IFN-γ (**G**), G-CSF (**H**), and IL-10 (**I**) in plasma (*n* = 10 dams per group) and concentrations of CXCL10 (**J**) and IL-10 (**K**) in amniotic fluid (*n* = 5–8 each). Data are presented as box plots where midlines indicate medians, boxes indicate IQR, and whiskers indicate minimum/maximum range. Symbols are values from individual dams and fetuses. Data were analyzed by Mann-Whitney *U* tests.

We next explored the mechanisms by which in vitro M2-polarized macrophages exert protection from preterm birth and neonatal loss. Maternal serum and amniotic fluid were obtained from dams administered intra-amniotic LPS with or without adoptive transfer of in vitro M2-polarized macrophages to quantify proinflammatory and antiinflammatory mediators (Figure 7A). Transfer of M2-polarized macrophages modulated the LPS-mediated induction of proinflammatory cytokines and chemokines in maternal blood, with reduced CCL2, CCL4, CXCL2, CXCL10, IL-5, IFN-γ, and G-CSF, as well as increased IL-10 (Figure 7, B–I). Transfer of M2-polarized macrophages also had antiinflammatory effects in the amniotic cavity, indicated by reduced CXCL10 and a tendency (although nonsignificant) toward increased IL-10 (Figure 7, J and K).

**Figure 8:**
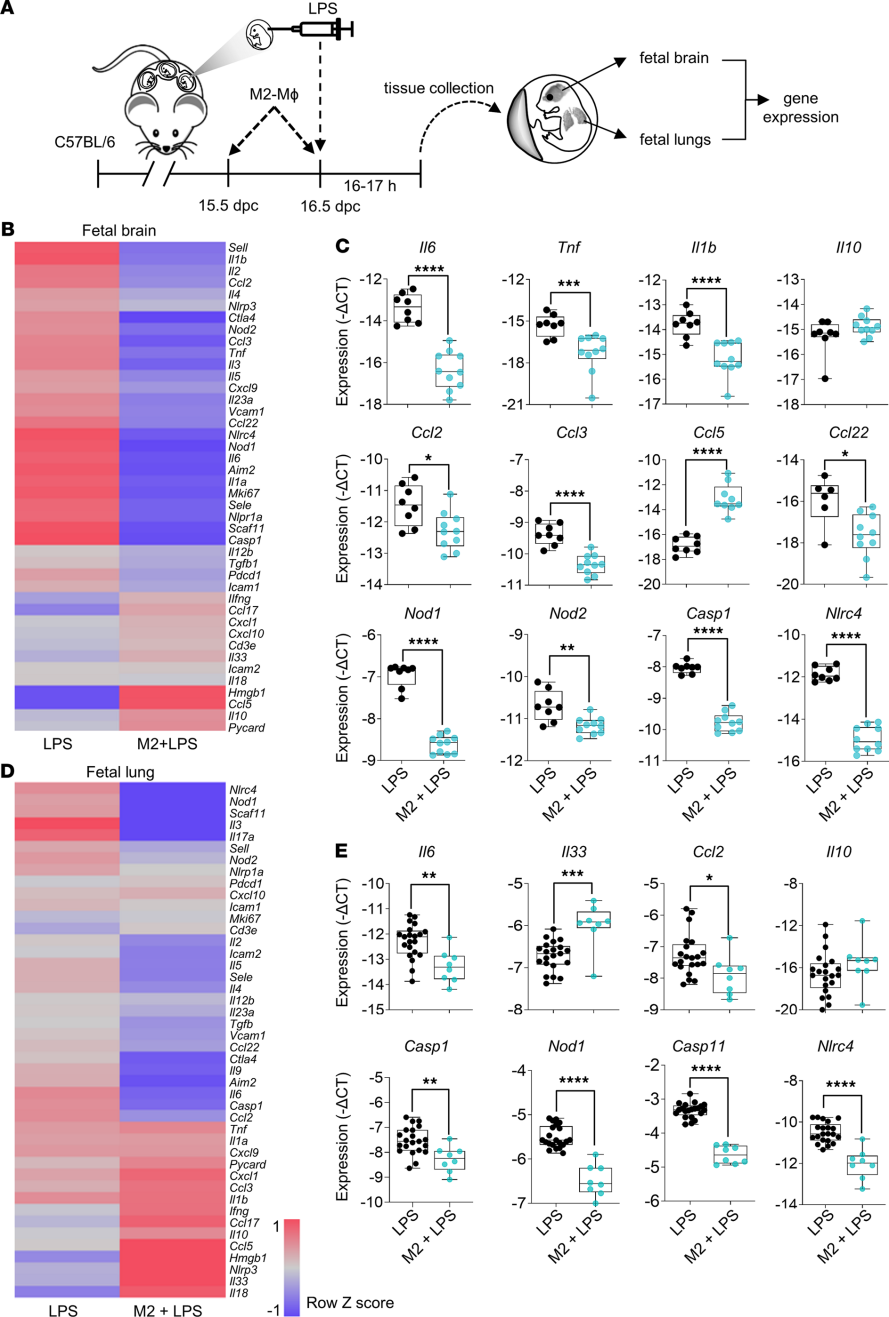
Adoptive transfer of M2-polarized macrophages to dams reduces inflammatory gene expression in fetal brain and lung after intra-amniotic administration of LPS. (**A**) M2-polarized macrophages (M2 Mɸ) or vehicle were i.v. administered on 15.5 dpc and 16.5 dpc to C57BL/6 dams followed by intra-amniotic injection with LPS on 16.5 dpc (M2+LPS). Control C57BL/6 dams were injected intra-amniotically with LPS only (LPS). Dams were euthanized 16 hours after LPS injection to collect fetal brain and lung for evaluation of gene expression. (**B**) Heatmap visualization of inflammatory gene expression in fetal brains from M2+LPS and LPS dams (*n* = 8–10 each). (**C**) Expression of *Il6*, *Tnf*, *Il1b*, *Il10*, *Ccl2*, *Ccl3*, *Ccl5*, *Ccl22*, *Nod1*, *Nod2*, *Casp1*, and *Nlrc4* in fetal brains. (**D**) Heatmap visualization of inflammatory gene expression in fetal lungs from M2+LPS and LPS dams (*n* = 8–21 each). (**E**) Expression of *Il6*, *Il33*, *Ccl2*, *Il10*, *Casp1*, *Nod1*, *Nod2*, and *Nlrc4* in fetal lungs. Data are presented as box plots with medians, IQRs, and minimum/maximum ranges. Symbols are values from individual fetuses. Data were analyzed by Mann-Whitney *U* tests. *P < 0.05, **P < 0.01, ***P < 0.001, ****P < 0.0001.

We also measured the expression of proinflammatory and antiinflammatory mediators in fetuses of dams that received intra-amniotic LPS with or without adoptive transfer of in vitro M2-polarized macrophages (Figure 8A). Proinflammatory gene expression was reduced in the fetal brain after transfer of M2-polarized macrophages (Figure 8B). Downregulated genes included proinflammatory mediators (*Il6*, *Tnf*, *Il1b*, *Il1a*, *Il2*, *Il3*, *Il23*), chemokines (*Ccl2*, *Ccl3*, *Ccl22*), inflammasome-related mediators (*Nod1*, *Nod2*, *Casp1*, *Nlrc4*, *Aim2*, *Nlrp1a*, *Casp11*), and cell adhesion molecules (*Sele*, *Sell*, *Vcam1*), while *Ccl5* was upregulated (Figure 8C and Supplemental Figure 11A). Similar changes were observed in the fetal lung (Figure 8D), where expression of the inflammatory mediators *Il6*, *Il5*, *Ccl2*, *Casp1*, *Nod1*, *Casp11*, *Nlrc4*, and *Aim2* decreased after transfer of M2-polarized macrophages (Figure 8E and Supplemental Figure 11B). In contrast, expression of *Il33* was increased in the fetal lung after transfer of M2-polarized macrophages (Figure 8E). In both the fetal brain and lung, *Il10* expression tended to increase but did not reach statistical significance (Figure 8, C and E).

Taken together, these findings demonstrated that M2-polarized macrophages can prevent intra-amniotic inflammation–induced preterm birth and adverse neonatal outcomes by reducing inflammatory responses in the maternal circulation, the amniotic cavity, and the fetal tissue.

## Discussion

Here, we report that women with spontaneous preterm birth exhibited a shift in the activation status of macrophages in the uterine decidua, with a reduced proportion of macrophages expressing markers of alternative activation. This suggested that macrophages might help sustain pregnancy, contrary to the common assumption that macrophages accelerate parturition. In support of a beneficial role, depletion of maternal CD11b^+^ myeloid cells from mice led to preterm birth and adverse neonatal outcomes. Depletion caused elevated proinflammatory mediators and increased activated leukocytes in the decidua, as well as increased neutrophils in the myometrium. Adoptive transfer of in vitro M2-polarized macrophages reduced preterm birth and improved neonatal survival in a model of intra-amniotic inflammation more effectively than nonpolarized macrophages. M2-polarized macrophages reduced proinflammatory mediators in the maternal blood and amniotic cavity and downregulated inflammatory gene expression in the fetal brain and lung. These data provide strong evidence that macrophages with antiinflammatory and homeostatic functions act to sustain uterine quiescence and fetal integrity in late gestation.

Our findings build on prior studies implicating homeostatic macrophages in fetal tolerance during early pregnancy (35, 36, 38, 62) and midgestation (34, 63) and expand on a report that systemic macrophage depletion from mice on 15 dpc caused fetal morbidity and mortality (28). However, earlier studies have not reported preterm birth or perinatal loss after macrophage depletion, suggesting the physiological roles of uterine macrophages depend on the gestational stage.

We found depletion of CD11b^+^ cells resulted in extensive loss of CD11b^+^F4/80^+^ cells but also caused a reduction in CD11b^+^CD11c^+^ myeloid DCs, as occurs when macrophages are depleted from *Cd11b^DTR/DTR^* mice in early gestation (64). Uterine macrophages and DCs have a close ontogenic relationship (47), share phenotypic characteristics (65), and operate within the broader context of innate and adaptive immune networks in the uterus and placenta (66–68). Activated T cells are implicated in the causal pathways accelerating preterm birth (61, 69, 70), and there is clear potential for myeloid DCs to attenuate T cell activation and phenotype in late gestation (27, 69). DCs with a regulatory phenotype are present in gestational tissues from implantation and are thought to promote materno-fetal tolerance by virtue of their eliciting type 2 T helper and Treg cell responses (71, 72). A shift away from this tolerogenic state occurs in decidual DCs near term, implying a role in the accompanying T cell changes (69). On the other hand, maintenance of a low decidual DC population may be critical for minimizing T cell priming to fetal antigens and the effector activity of sporadic infiltrating T cells (47). Considering the potential for DCs and macrophages to regulate T cell phenotypes, it was possible that CD11b^+^ cell depletion would result in expanded effector T cell populations that in turn could mediate fetal damage. However, we did not identify any change in the total number of decidual CD4^+^ or CD8^+^ T cells after CD11b^+^ cell depletion. Nevertheless, we cannot exclude a possible contribution of myeloid DCs to the causal events of preterm birth after DT administration in *Cd11b^DTR/DTR^* mice, for example, through attenuating the balance of T cell regulatory or inflammatory phenotypes.

CD11b^+^ myeloid cell depletion also induced elevated circulating neutrophils as well as neutrophil influx into the uterine myometrium. Previous studies in *CD11b^DTR/DTR^* mice show macrophage loss promotes granulopoiesis (73, 74), secondary to clearance of apoptotic neutrophils (75). Neutrophil recruitment is a consistent precursor of uterine activation, myometrial contractility, and preterm birth (22, 23). Previous reports show that women who undergo spontaneous preterm birth have higher circulating neutrophils than women with iatrogenic preterm birth (76). Limiting neutrophil influx into myometrial tissues during late gestation could thus be one mechanism by which homeostatic macrophages assist in maintaining uterine quiescence.

CD11b^+^ cell depletion substantially increased expression of inflammatory mediators in decidual tissues and caused activation of resident leukocytes, particularly NK cells. NK cells are abundant in the materno-fetal interface in the third trimester (77), and their increased activation is associated with preterm birth (78, 79). M2 macrophages are a rich source of IL-10 (30) and potentially suppress uterine NK cells through IL-10 release (80), as demonstrated by studies in *Il10*-null mutant mice, which exhibit heightened susceptibility to preterm birth associated with uterine NK cell activation (49, 81). A regulatory effect of macrophages on uterine NK cells in late gestation is in line with several studies demonstrating that uterine NK cell activation, and other perturbations to pregnancy tolerance, lead to preterm birth and adverse neonatal outcomes (24, 27, 49). Decidual stromal cells undergo epigenetic modification to prevent certain effector immune cells from accessing the placental interface, and this is reversed in response to proinflammatory stimuli (82, 83). We speculate that homeostatic macrophages might contribute to enforcing this epigenetic state, but this remains to be investigated.

Finding suitable therapies for the prevention or treatment of spontaneous preterm birth remains a major clinical challenge (13, 84, 85). Currently, the only effective intervention is administration of vaginal progesterone to women with a short cervix (55). Progesterone is believed to prolong uterine quiescence through potent antiinflammatory effects (51). In the current study, progesterone effectively suppressed uterine activation but not fetal inflammatory injury. This may be due to the vulnerability of developing fetal tissues to inflammatory mediators (15) and/or their limited capacity for progesterone signaling (50). Given that plasma progesterone was not affected by macrophage depletion, these data indicate that macrophages sustain late gestation through a mechanism other than via modulating progesterone synthesis, in contrast to a model of midgestation pregnancy loss induced by activation of the innate immune system (52).

To understand how macrophages suppress preterm birth and alleviate adverse perinatal outcomes, we first tested whether adoptive transfer of nonpolarized macrophages could mitigate the detrimental effects seen after depletion. Restoration of macrophages extended gestation length, prevented preterm birth, and improved neonatal survival and postnatal growth impairment. This showed that macrophages are primarily responsible for the adverse outcomes elicited by CD11b^+^ cell depletion in *CD11b^DTR/DTR^* mice and confirmed their significance in protecting late gestation, but raised the question of whether the phenotype of macrophages is critical. We observed that, as gestation progressed, decidual macrophages released increasing IL-10 (a hallmark antiinflammatory product of M2 macrophages and inhibitor of proinflammatory IL-12 secretion) (56, 57), consistent with prior demonstrations that M2 macrophages in the uterine decidua produce IL-10 (18, 35). An exploratory experiment showed strongly elevated decidual *Il6*, *Irf1*, and *Cxcl3* expression after macrophage depletion. These results pointed to M2-like activity in uterine macrophages in late gestation, as occurs earlier in pregnancy (35). The efficacy of unpolarized and in vitro M2-polarized macrophages was then directly compared in a model of intra-amniotic LPS–induced preterm birth (45, 60), which more closely reflects the human pathophysiology of premature labor. Consistent with our hypothesis, M2-polarized macrophages demonstrated superior protective activity over nonpolarized macrophages, with only M2 macrophages effective in reducing the incidence of preterm birth and neonatal mortality induced by intra-amniotic LPS.

The reason that unpolarized macrophages were effective in the *Cd11b^DTR/DTR^* model but not the intra-amniotic LPS model most likely relates to the proinflammatory insult of LPS. Presumably, M2 macrophages were more potent suppressors of inflammation than BMDMs by virtue of their phenotype being further skewed and more stably embedded. In another study, macrophage depletion using anti-F4/80 antibody suppressed preterm birth caused by intravaginal LPS (54), implying that insufficiently stable homeostatic macrophages can convert to proinflammatory effector activity in response to elevated TLR signaling. M2 macrophages generated in vitro may be better able to retain and express an antiinflammatory effector function and be less responsive to local cues than unpolarized BMDMs.

We and others have shown that biologic drugs targeting inflammatory cytokines can prevent preterm birth and adverse neonatal outcomes by dampening systemic and local inflammatory responses (44, 45, 86–88). Consistent with a similar action, we showed that M2-polarized macrophages infiltrated the materno-fetal interfaces after transfer and modulated cytokine production locally in gestational tissues, as reflected in the amniotic cavity, as well as systemically in the maternal peripheral blood. Importantly, the transferred M2-polarized macrophages reduced fetal inflammatory responses induced by LPS in the fetal brain and lungs, indicating that this cellular approach is active in the fetal compartment and has comparable efficacy to other antiinflammatory interventions that alleviate fetal inflammation (45, 87, 88). However, M2 macrophages were not 100% effective in rescuing neonates born to LPS-treated dams and some did not survive. This was most likely due to less-than-complete macrophage reconstitution, given the technical limitation on delivering sufficient cells to fully recapitulate the steady-state condition, and evidence that uterine and placental cells were not fully reconstituted by adoptive transfer. These data imply that the late-gestation fetus is highly dependent on maternal homeostatic macrophages for protection from inflammatory injury, to a greater degree than the quiescent uterus. Consistent with this, fetal inflammatory injury has previously been demonstrated under conditions of mild uterine inflammation that are insufficient to cause preterm birth (88, 89). The rapidly developing fetal brain, lung, and gastrointestinal tract are thought to be highly vulnerable to effects of inflammatory mediators that can program permanent structural changes (14, 15).

We found M2 macrophages attenuated fetal brain and lung expression of inflammasome components, resonating with an emerging role for inflammasome assembly in gestational disorders and fetal tissue injury (12, 90, 91). A feature of treatment with M2-polarized macrophages was upregulated expression of IL-33 in the fetal lung. This cytokine is a crucial immune modulator that shapes type 1, type 2, and regulatory immune responses (92). The maternal and/or fetal tissue compartment in which passively transferred macrophages act to attenuate fetal cytokine expression is unclear. A high abundance of transferred cells in the placenta implies a function in suppressing fetal proinflammatory activation through direct or indirect effects on the placental immune network. Here, maternal macrophages could suppress placental inflammatory injury and limit placental transfer of inflammatory mediators (15). This likely reflects accumulation of transferred macrophages in maternal blood spaces, which seems more probable than migration across the trophoblast barrier into placental villous tissue or the fetus itself. However, further investigation of the site of action is required, as fetal trafficking of maternal cells is reported to occur (93), and maternal bone marrow–derived mesenchymal stem cells are thought to alleviate perinatal inflammatory injury to the fetal brain by this route (94).

These findings provide solid evidence that maternal macrophages exert homeostatic effects on maternal and fetal tissues during late gestation and that, in the event of deficiency, their restoration can be an effective strategy to dampen maternal and fetal inflammation. The feasibility of targeting M2 macrophages for clinical benefit in pregnant women at risk of preterm birth and/or fetal inflammatory injury therefore warrants consideration — especially as our data imply that macrophage therapies have potential for clinical effects distinct from those of progesterone, the most effective existing therapy for women at risk of preterm labor (95). M2-polarized macrophages generated in vitro have been investigated for therapeutic applications in cancer, and clinical efficacy with minimal side effects was reported (96). A recent clinical trial reported significant neurological improvement after intrathecal transfer of M2-polarized autologous macrophages to stroke patients (97). These investigations provide a rationale for considering adoptive transfer of autologous M2-polarized macrophages in appropriately selected pregnant women at risk for delivering preterm. Whether this approach is appropriate for protection against the range of inflammatory stimuli implicated in spontaneous preterm birth, including sterile as well as microbial agents (5–7), will be important to consider.

Future research is required to define the specific mechanisms whereby homeostatic macrophages regulate susceptibility to uterine and fetal inflammation. In particular, given the significance of fetal immune activation in triggering premature labor (70, 98, 99), it will be critical to define whether insufficiency or instability in maternal homeostatic macrophages contributes to initiating fetal and placental immune activation, as would be predicted in line with a paramount role for maternal regulation of inflammatory cues in preterm labor (100).

In conclusion, this study provides descriptive and mechanistic evidence that macrophages play a central homeostatic role in pregnancy maintenance in late gestation. The requirement for macrophages to exert protective, antiinflammatory functions in late gestation is reminiscent of homeostatic actions in other tissue settings (29, 30). Collectively, the findings reported in this study support the evaluation of M2-polarized macrophages as a therapeutic target in women with threatened spontaneous preterm birth and to protect infants from poor neonatal outcomes caused by inflammatory exposures in utero.

## Methods

### Human tissue and decidual leukocyte analysis.

Human decidual tissue samples were collected at Hutzel Women’s Hospital in the Detroit Medical Center, Detroit, Michigan, USA, from women with spontaneous preterm birth, iatrogenic preterm birth, or term delivery (demographic and clinical characteristics shown in Table 1 and Supplemental Table 1). Leukocytes were isolated from decidual tissues of each study group by using a Miltenyi Biotec gentleMACS Dissociator followed by flow cytometry analysis. Further details are provided in Supplemental Methods.

### Mice and experimental treatments.

Female *Cd11b^DTR/DTR^* mice (Tg[ITGAM-DTR/EGFP]34Lan, provided by Richard Lang, University of Cincinnati, Cincinnati, Ohio, USA) (43) and *Cd11b^WT/WT^* (WT FVB/N) were mated with BALB/c males (both Animal Resources Centre, Canning Vale, Western Australia, Australia), and the presence of a vaginal plug indicated 0.5 dpc. *Cd11b^DTR/DTR^* dams were administered 25 ng/g of DT on 16 dpc or 17 dpc to deplete CD11b^+^ cells. Control *Cd11b^DTR/DTR^* dams were given 200 μL PBS, and *Cd11b^WT/WT^* dams were given 25 ng/g DT. Some *Cd11b^DTR/DTR^* and *Cd11b^WT/WT^* dams also received progesterone or BMDMs. C57BL/6 dams received an ultrasound-guided intra-amniotic injection of 100 ng LPS. After treatments, dams were monitored to identify delivery time or euthanized for tissue recovery. Preterm birth was defined as delivery within 48 hours of intervention, and the rate was calculated as the proportion of the total number of pregnant mice. Pup survival and weight were evaluated at birth, at 12–24 hours after birth, and at 8 and 21 days of age. Further details are provided in Supplemental Methods.

### Isolation, differentiation, and adoptive transfer of BMDMs.

Bone marrow from *Cd11b^WT/WT^* mice was incubated in media supplemented with 20% L929 conditioned media as described (32). C57BL/6 bone marrow cells were incubated with 10 ng/mL of recombinant CSF-1 (BioLegend, 576402) for 7 days, then with 10 ng/mL each of recombinant IL-4 (BioLegend, 574302) and IL-13 (BioLegend, 575902) (M2-polarized macrophages), or fresh IMDM plus 10% FBS and 10 ng/mL CSF1 (unpolarized macrophages), for 2 days. Some mice were administered GFP^+^ M2-polarized BMDMs from C57BL/6-Tg(CAG-EGFP)131Osb/LeySop mice to allow tracking of passively transferred macrophages recruited into gestational tissues. Further details are provided in Supplemental Methods.

### Immunohistochemistry and immunophenotyping of murine leukocytes.

Whole uterus (myometrium and endometrium) and placenta were collected from *Cd11b^DTR/DTR^* dams 24 hours after administration of DT or PBS on 16 dpc, and macrophages were detected with rat anti–mouse F4/80 by immunohistochemistry. Single-cell suspensions were prepared from the uterine myometrium, uterine decidua, placenta, and fetal liver, as previously described (101), from *Cd11b^DTR/DTR^* or *Cd11b^WT/WT^* dams 24 hours after administration of DT or PBS on 16 dpc, and leukocytes were analyzed by flow cytometry. Further details are provided in Supplemental Methods.

### Quantification of progesterone, cytokine secretion by myometrial/decidual macrophages, cytokine concentrations in maternal plasma and amniotic fluid, and gene expression.

Peripheral blood plasma progesterone was measured by using an enzyme-linked immunoassay (Mouse/Rat Progesterone Kit, ALPCO Diagnostics, 55-PROMS-E01), following the manufacturer’s instructions. Supernatants from myometrial and decidual tissue macrophages, sorted by positive selection of F4/80^+^ cells, were analyzed for IL-10, IL-12p70, TNF, and TGF-β concentrations by using Quantikine ELISA Kits (R&D Systems, Bio-Techne). Maternal plasma and amniotic fluid cytokines were measured by using the ProcartaPlex Mouse Cytokine & Chemokine Panel 1A 36-plex (Invitrogen, Thermo Fisher Scientific) and Luminex 100 SystemFill. cDNA from decidual tissue collected 24 hours after intervention was analyzed by using QIAGEN RT² Profiler Mouse Innate & Adaptive Immune Response and RT² Profiler Mouse Inflammatory Response & Autoimmunity PCR arrays (Supplemental Table 4). Interaction network analysis was performed by using STRING v.10.0 software (102). Fetal brain and lung cDNA were analyzed by Fluidigm BioMark System for high-throughput RT-qPCR with TaqMan gene expression assays (Applied Biosystems, Thermo Fisher Scientific; Supplemental Table 5). Further details are provided in Supplemental Methods.

### Statistics.

Statistical analyses were performed by using SPSS v19.0 (IBM) and Prism v8.3.0 (GraphPad). For human demographic data, the group comparisons were performed by using the Mann-Whitney *U* test for continuous variables, Fisher’s exact test for binary nominal variables, and χ^2^ test for categorical nominal variables. For mouse data, Fisher’s exact test was performed to evaluate preterm birth rate and other categorical data, and Kaplan-Meier survival curves were used to plot and compare neonatal survival data (Mantel-Cox test). A Shapiro-Wilk test was performed to determine whether data were normally distributed. Comparisons among multiple groups were performed by using 1-way ANOVA and a post hoc 2-tailed *t* test, by using the 2-stage linear step-up procedure of Benjamini, Krieger, and Yekutieli to correct for multiple comparisons in normally distributed data sets, or the Kruskal-Wallis test followed by Mann-Whitney *U* test for non-normally distributed data sets. For RT-qPCR arrays, the –ΔCt values were normalized by calculating the *z* score of each gene, and then heatmaps were created to represent the mean of the *z* score of –ΔCt and hierarchical clustering by using Euclidian distance. *P* values were considered significant when *P* was less than 0.05.

### Study approval.

The collection and utilization of human biological materials for research purposes were approved by the IRBs of Wayne State University (WSU) and the *Eunice Kennedy Shriver* National Institute of Child Health and Human Disease (WSU IRB 082403MP2F and WSU IRB 110605MP2F). All participating women provided written informed consent prior to sample collection.

*Cd11b^DTR/DTR^* mice were cared for and used in experiments as detailed in the National Health and Medical Research Council of Australia (NHMRC) Australian Code of Practice for the Care and Use of Animals for Scientific Purposes with ethics approval from the Animal Ethics Committee, University of Adelaide (ethics IDs: M-2010-055 and M-2012-153). A Genetically Modified Organisms Dealing Authorization was obtained from the Institutional Biosafety Committee, University of Adelaide (ID: 10511). The use of C57BL/6, FVB/V, and C57BL/6-Tg(CAG-EGFP)131Osb/LeySop mice in experiments was approved by the IACUC at WSU (protocols A-09-08-12, A-07-03-15, and A-18-03-0584). The authors adhered to the NIH *Guide for the Care and Use of Laboratory Animals* (National Academies Press, 2011).

## Author contributions

SAR and NGL designed the study; NGL, VGF, PYC, HMG, KRD, and MTB performed research; NGL, SAR, VGF, PYC, and HMG analyzed data; RR provided clinical materials; NGL and SAR wrote the paper.

## Supplementary Material

Supplemental data

## Figures and Tables

**Table 1:**
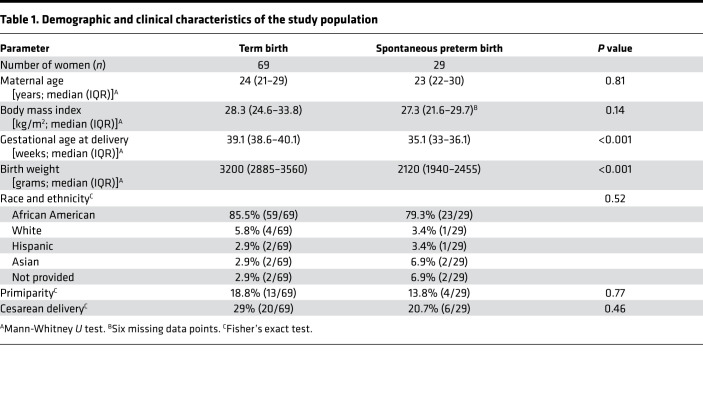
Demographic and clinical characteristics of the study population

## Version 1 10/08/2021

Electronic publication
