# Supplementary material for: Macrophages exert homeostatic actions in pregnancy to protect against preterm birth and fetal inflammatory injury
Source: JCI Insight. 2021 Oct 8;6(19):e146089. doi: 10.1172/jci.insight.146089 (PMC8525593; doi:10.1172/jci.insight.146089)
Supplement: Supplemental data [file jciinsight-6-146089-s126.pdf]

Supplementary Information for

Macrophages exert homeostatic actions in pregnancy to protect against preterm birth and fetal inflammatory injury

Authors: Nardhy Gomez-Lopez[1-4], Valeria Garcia-Flores[2,3], Peck Yin Chin[1], Holly M. Groome[1], Melanie T. Bijland[1], Kerrilyn R. Diener[1,5], Roberto Romero[2,6-10], Sarah A. Robertson[1]

[1]Robinson Research Institute and Adelaide Medical School, The University of Adelaide, Adelaide, South Australia, AU
[2]Perinatology Research Branch, Division of Obstetrics and Maternal-Fetal Medicine, Division of Intramural Research, *Eunice Kennedy Shriver* National Institute of Child Health and Human Development, National Institutes of Health, U.S. Department of Health and Human Services (NICHD/NIH/DHHS); Bethesda, Maryland, and Detroit, Michigan, USA
[3]Department of Obstetrics and Gynecology, Wayne State University School of Medicine, Detroit, Michigan, USA
[4]Department of Biochemistry, Microbiology and Immunology, Wayne State University School of Medicine, Detroit, Michigan, USA
[5]University of South Australia Cancer Research Institute, Clinical and Health Sciences, University of South Australia, Adelaide, South Australia, AU
[6]Department of Obstetrics and Gynecology, University of Michigan, Ann Arbor, MI, USA.
[7]Department of Epidemiology and Biostatistics, Michigan State University, East Lansing, MI, USA
[8]Center for Molecular Medicine and Genetics, Wayne State University, Detroit, MI, USA.
[9]Detroit Medical Center, Detroit, MI, USA
[10]Department of Obstetrics and Gynecology, Florida International University, Miami, FL, USA

Corresponding author:
Sarah A Robertson, PhD, Robinson Research Institute, Adelaide School of Medicine, University of Adelaide, Adelaide Health and Medical Sciences Building, North Terrace, Adelaide SA 5005, Australia, T: +61 8 8313 4094, E-mail: sarah.robertson@adelaide.edu.au
Nardhy Gomez-Lopez, PhD, Department of Obstetrics and Gynecology, Wayne State University, School of Medicine, Perinatology Research Branch, NICHD/NIH/DHHS, 275 E Hancock St, Detroit, Michigan 48201 USA, Tel: 313-577-8904, Email: nardhy.gomez-lopez@wayne.edu

This PDF file includes:

SI Materials and Methods
Supplementary Figures 1 to 11
Supplementary Tables 1 to 5

SI Materials and Methods

Human subjects, clinical specimens, and definitions

Human decidual tissues samples were collected at Hutzel Women's Hospital in the Detroit Medical Center, Detroit, MI, USA, in partnership with Wayne State University School of Medicine and the Perinatology Research Branch, an intramural program of the *Eunice Kennedy Shriver* National Institute of Child Health and Human Development, National Institutes of Health, US Department of Health and Human Services (NICHD/NIH/DHHS), Detroit, MI, USA. The study groups included women who delivered at term, women with spontaneous preterm birth, and women with iatrogenic preterm birth. Term birth was defined as delivery after 37 weeks of gestation whereas preterm birth occurred before 37 weeks of gestation. Gestational age was determined by the date of the last menstrual period and confirmed by ultrasound examination. Spontaneous preterm birth was defined as delivery following the spontaneous onset of labor. Patients with multiple births or neonates that had congenital or chromosomal abnormalities were excluded from this study. The demographic and clinical characteristics of the study populations are shown in Table 1 and Supplementary Table 1.

Isolation of human decidual leukocytes

Leukocytes were isolated from decidual tissues of each study group, as previously described (1). Briefly, the decidua basalis and the decidua parietalis were homogenized using a gentleMACS Dissociator (Miltenyi Biotec, San Diego, CA, USA) in StemPro Accutase Cell Dissociation Reagent (Life Technologies, Carlsbad, CA, USA) and incubated for 45 minutes (min) at 37°C with gentle agitation. After incubation, tissues were washed in phosphate-buffered saline (PBS) (Life Technologies) and filtered through a 100 µm cell strainer (Falcon; Corning Life Sciences, Durham, NC, USA). The resulting cell suspension was centrifuged at 300 x g for 10 min at 4°C. Decidual leukocytes were then separated by density gradient using Ficoll-Paque Plus (GE Healthcare Biosciences, Uppsala, Sweden). Cells collected from the mononuclear layer of the density gradient were washed with PBS and immediately used for immunophenotyping.

Immunophenotyping of human decidual macrophage subsets

Isolated decidual mononuclear cells were incubated with 20 µL of human FcR blocking reagent (Miltenyi Biotec) in 80 µL of stain buffer (Cat#554656; BD Biosciences, San Jose, CA, USA) for 10 min at 4°C, then incubated with fluorochrome-conjugated anti-human monoclonal antibodies for 30 min at 4°C in the dark (Supplementary Table 2). After extracellular staining, the cells were washed with PBS to remove excess antibody, resuspended in 0.5 mL stain buffer, and acquired using the BD LSR II Flow Cytometer (BD Biosciences) and BD FACSDiva 6.0 software (BD Biosciences). For intracellular staining, the cells were then fixed and permeabilized using the BD Cytofix/Cytoperm™ Fixation and Permeabilization Solution (BD Biosciences). Next, the cells were washed with BD Perm/Wash™ Buffer (BD Biosciences), re-suspended in 50 µL of the same buffer, and stained with intracellular antibodies for 30 min at 4°C in the dark (Supplementary Table 2). Finally, the stained cells were washed with BD Perm/Wash™ Buffer, re-suspended in 0.5 ml stain buffer, and acquired using the BD™ LSR II Flow Cytometer and BD™ FACSDiva 6.0 software. Data analysis was performed using FlowJo software version 10 (FlowJo, Ashland, OR, USA).

Mice

$Cd11b^{DTR/DTR}$ mice expressing the simian diphtheria toxin receptor (DTR) driven by the *Cd11b* promoter on an inbred FVB/N background (Tg[ITGAM-DTR/EGFP]34Lan) (2, 3) were provided by Richard Lang (University of Cincinnati, Cincinnati, Ohio, USA). $Cd11b^{DTR/DTR}$ and $Cd11b^{WT/WT}$ (wild-type FVB/N) mice were housed and bred under specific pathogen-free conditions at the University of Adelaide Medical School Animal House on a 12 hour (h) light/12 h dark cycle. Eight- to twelve-week-old $Cd11b^{DTR/DTR}$ and $Cd11b^{WT/WT}$ females were mated with

BALB/c males and examined the following morning for the presence of a vaginal plug, which indicated 0.5 days *post coitum* (dpc). C57BL/6, FVB/N, and C57BL/6-Tg(CAG-EGFP)131Osb/LeySop mice were purchased from The Jackson Laboratory (Bar Harbor, ME, USA). Mice were bred in the animal care facility at the C.S. Mott Center for Human Growth and Development (Wayne State University, Detroit, MI, USA) and housed on a 12 h light/12 h dark cycle. Eight- to twelve-week-old C57BL/6 or FVB/N females were mated with males of proven fertility and examined daily between 8:00 and 9:00 a.m. for the presence of a vaginal plug. After observation of a vaginal plug, female mice were removed from the mating cages and housed separately. A weight gain ≥2 grams at 12.5 dpc confirmed pregnancy.

Experimental treatments in pregnant mice and perinatal outcome variables

Cd11b$^{DTR/DTR}$ dams were administered 25 ng/g of diphtheria toxin (DT) (in 200 µl PBS, i.p.) from *Corynebacterium diphtheriae* (Sigma, St. Louis, MO, USA) on either 16.0 dpc (15.5 dpc at 5-6 pm) or 17.0 dpc (16.5 dpc at 5-6 pm) to deplete CD11b^{+} cells. Control *Cd11b*$^{DTR/DTR}$ dams were administered 200 µL PBS and *Cd11b*$^{WT/WT}$ females were administered 25 ng/g of DT in 200 µL PBS. A group of *Cd11b*$^{DTR/DTR}$ and *Cd11b*$^{WT/WT}$ dams was also administered progesterone (Sigma) (200 ng/100 µl sesame oil) or sesame oil vehicle control (Sigma) on 16.0 dpc, 17.0 dpc, and 18.0 dpc. The endotoxin content of DT was confirmed to be <0.25 EU/ml (25-50 pg/ml) by Limulus amebocyte assay (E-Toxate, Sigma Aldrich).

Ultrasound-guided intra-amniotic injection of LPS was performed on 16.5 dpc C57BL/6 mice, as previously described (4-6). Briefly, dam anaesthesia was induced with 2–3% isoflurane (Aerrane, Baxter Healthcare Corporation, Deerfield, IL, USA) and 1–2 L/min of oxygen, then maintained with 1.5–2% isoflurane and 1.5–2 L/min of oxygen. Dams were positioned on a heating pad and abdominal hair removed. Body temperature was monitored by rectal probe (Visual Sonics, Toronto, ON, Canada) and maintained at 37 ± 1°C, and respiratory and heart rates were monitored by electrodes embedded in the heating pad. Using an ultrasound probe fixed and mobilized with a mechanical holder, ultrasound guided intra-amniotic injection of LPS (*Escherichia coli* O111: B4, Sigma)(100 ng in 25 µL of sterile PBS, or 25 µL of PBS alone for controls) was performed in each amniotic sac using a 30G needle (Becton Dickinson) stabilized by a mechanical holder (VisualSonics). Following treatments, dams were either monitored by infrared camera (Sony, Tokyo, Japan) until delivery or euthanized for tissue recovery at specific time points in late gestation, as detailed below.

Preterm birth was defined as delivery within 48 h of intervention (CD11b^{+} myeloid cell depletion or LPS administration): ≤18.0 dpc (Figure 2B & Supplementary Figure 9C) or <18.5 dpc (Figure 4B, Figure 6C), and the rate was calculated as proportion of total number of pregnant mice. Gestational length was calculated as the length of time from the presence of the vaginal plug until the appearance of the first pup in the cage bedding. Neonatal survival (% pups born) was calculated at birth, at 12-24 h after birth, and at 8 and 21 days of age. Pup weights were recorded at 12-24 h after birth, as well as 8 and 21 days of age.

Isolation, differentiation, and adoptive transfer of bone marrow-derived macrophages

For reconstitution experiments in *Cd11b*$^{DTR/DTR}$ mice, bone marrow was collected from *Cd11b*$^{WT/WT}$ mice and treated with red blood cell lysis buffer. Cells were resuspended in complete RPMI medium (cRPMI, 10% FBS, 100 U/mL penicillin, 100 mg/mL streptomycin, 1% L-glutamate) supplemented with 20% L929 conditioned media as a source of CSF-1 and seeded at 1 x 10^{6} cells/ml, as described (7). Macrophages were collected on day 6 for adoptive transfer experiments after repeated washing with ice-cold PBS using StemPro Accutase Cell Dissociation Reagent (Life Technologies). Approximately 5–10 x 10^{6} cells were resuspended in 250 µl RPMI for intravenous (i.v.) injection into each dam on both 14.5 and 16.5 dpc, prior to i.p. administration of DT, or PBS on 16.5 dpc. This number was the maximum number of cells that could feasibly be transferred by tail vein injection, and was judged sufficient to partially repopulate a *CD11b*$^{DTR/DTR}$ mouse after DT administration, based on

estimates of ~3 x 10^5 CD11b$^+$F4/80$^+$ cells/ ml in peripheral blood, and ~1 x 10^6 CD11b$^+$F4/80$^+$ cells/ g of uterus (Fig. 2B).

For experiments in C57BL/6 mice, bone marrow-derived cells from C57BL/6 donors were treated with red blood cell lysis buffer and cultured in IMDM medium (Thermo Scientific) supplemented with 10% FBS (Invitrogen, Carlsbad, CA, USA) and 10 ng/mL of recombinant CSF1 (Cat#576402; BioLegend, San Diego, CA, USA), and incubated at 37°C with 5% CO_2 for 7 days. On day 7, the culture medium was replaced with fresh IMDM medium + 10% FBS and 10 ng/mL each of recombinant IL-4 (Cat#574302, BioLegend) and IL-13 (Cat#575902, BioLegend), or fresh IMDM medium + 10% FBS and 10 ng/mL CSF1. On day 8 of culture, 14-18 hours later, macrophages were collected for adoptive transfer. Either M2-polarized BMDM or non-polarized BMDM (2 – 3 x 10^6) were re-suspended in 100 µL sterile PBS for i.v. injection on 15.5 or 16.5 dpc, prior to the intra-amniotic administration of lipopolysaccharide (LPS), or PBS. Flow cytometry was performed to quantify expression of phenotype markers Egr-2 and Ym1/2 in both BMDM preparations (Supplementary Table 3). Some mice were administered GFP$^+$ M2-polarized BMDM to allow tracking of passively transferred macrophages recruited into gestational tissues. BMDM were recovered from C57BL/6-Tg(CAG-EGFP)131Osb/LeySop mice, cultured and administered to recipients, as described above. Leukocytes were isolated from myometrial, decidual, and placental tissues as previously described, and flow cytometry was performed to determine the numbers of GFP$^+$F4/80$^+$ cells in each tissue as detailed below.

Immunohistochemistry

Whole uterus (myometrium and endometrium) and placenta were collected from *Cd11b$^{DTR/DTR}$* dams 24 h after administration of DT or PBS on 16.0 dpc. Tissue sections (5 µm thick) were cut and mounted on glass microscope slides. The sections were blocked with 15% normal mouse serum (NMS) and 15% normal rabbit serum for 30 min at 37ºC followed by overnight incubation with rat anti-mouse F4/80 in PBS (1:100 dilution), 1.5% NMS, and 1.5% normal rabbit serum (eBioscience, San Diego, CA, USA) at 4ºC in a humidified chamber. Afterwards, the tissues were incubated with biotinylated rabbit anti-rat IgG (1:200 dilution) for 40 min at room temperature (Vector Laboratories, Burlingame, CA, USA), followed by a 30 min incubation with Elite ABC reagent from the VECTASTAIN ABC Elite kit (Vector Laboratories). Horseradish peroxidase was localized with diaminobenzidine tetrachloride (DAB) (DAKO, Glostrup, Denmark), and tissues were counterstained with hematoxylin (Sigma). Negative control sections were incubated either with irrelevant primary antibody, the secondary antibody alone, or no antibody. There was no positive staining identified in the control slides. Within the labyrinth zone of the placenta, F4/80$^+$ cells in the intervillous space containing maternal blood, identified by their characteristic structure and the presence of red blood cells, were considered macrophages of maternal origin. F4/80$^+$ cells within the interstitial compartment, exhibiting structural features typical of villous tissue with a syncytiotrophoblast lining, were considered macrophages of fetal origin (Hofbauer cells) (8, 9).

Isolation of murine leukocytes for immunophenotyping

Maternal and fetal tissues were collected from *Cd11b$^{DTR/DTR}$* dams 24 h after administration of DT or PBS on 16.0 dpc, and *Cd11b$^{WT/WT}$* dams 24 h after administration of 25 ng/g of DT on 16.0 dpc, and leukocytes were isolated as previously described (10). Dams were weighed and anesthetized with avertin [tribromoethanol dissolved in amylene hydrate and distilled water] (Sigma). Peripheral blood was recovered by cardiac puncture and placed in tubes containing heparin sodium (Sigma). Mice were then euthanized by cervical dislocation, and the uterine myometrium, uterine decidua, placenta, and fetal liver were collected, rinsed in PBS, pooled (3-4 tissues, respectively), and gently dissociated in StemPro Accutase Cell Dissociation Reagent (Life Technologies) using scissors. After incubation at 37ºC for 40 min, cells were filtered using a 100 µm cell strainer (Becton Dickinson, Franklin Lakes, NJ, USA) and washed with FACS buffer (0.1% bovine serum albumin and 0.05%

sodium azide in PBS). Cell debris was removed by centrifugation in 1 mL of fetal calf serum, followed by washing with 500 µL of FACS buffer.

Immunophenotyping of murine leukocytes

Immediately after isolation, leukocyte cell pellets from the blood, myometrium, decidua, placenta, and fetal liver were re-suspended in FACS buffer and pre-incubated with Fc Block (Becton Dickinson, clone 2.4G2) for 10 min on ice and subsequently incubated with specific fluorochrome-conjugated anti-mouse antibodies shown in Supplementary Table 3. Red blood cells were lysed using RBC lysis buffer (150 mM ammonium chloride, 10 mM potassium bicarbonate and 0.1 mM EDTA, pH 7.2). The lineage and phenotype of viable leukocytes [4′,6-diamidino-2-phenylindole (DAPI)$^{+}$CD45^{+} cells] were analyzed using combinations of antibodies against CD45, CD11b, F4/80, CD11c, MHC class II, Ly6G, CD3, CD4, CD8, CD49b, and CD69 according to gating strategies shown in Supplementary Figures 6A, 7 and 8A. Total numbers of leukocyte subsets were determined using CountBright Absolute Counting Beads (Molecular Probes, Invitrogen, Eugene, OR, USA). The term 'cells/ml' refers to the number of cells per ml of FACS buffer, at the time of flow cytometry analysis, and is directly proportional to the number of cells/uterus. The number of cells per ml was calculated using the formula provided by CountBrightTM Absolute Counting Beads (Molecular Probes). This formula considers the number of cells, number of beads, and volume in which cells were resuspended. For tissues, cells were obtained after dissociation of ~30-50 mg of tissue resuspended in 1 ml of FACS buffer. For blood, 100 uL of blood was utilized in all experiments and white blood cells were resuspended in 1 mL of FACS buffer. The analysis and preparation of images were performed using FlowJo Software Version 10.

Gene expression analysis in murine decidual tissue

Uterine decidual tissue was collected from *Cd11b*$^{DTR/DTR}$ dams 24 h after administration of DT or PBS on 16.0 dpc. Total RNA was extracted from each sample with Trizol, following the manufacturer's instructions. RNA concentration and purity were assessed with the NanoDrop 1000 spectrophotometer (Thermo Scientific, Wilmington, DE, USA) and RNA integrity was evaluated using agarose gels. Complementary (c) DNA was synthesized using the RT^{2} First Strand Kit (QIAGEN, Hilden, Germany). A single pooled sample was generated for each group (n = 7-8 dams/pool). The RT^{2} Profiler Mouse Innate & Adaptive Immune Response PCR Array (Cat#PAMM-052ZA-2, QIAGEN) and RT^{2} Profiler Mouse Inflammatory Response & Autoimmunity PCR Array (Cat#PAMM-077ZA-2, QIAGEN) were used for mRNA expression profiling and performed using RT^{2} SYBR Green ROX qPCR MasterMix (QIAGEN) on a 7500 Fast Real-Time PCR System (Applied Biosystems, Life Technologies, Foster City, CA, USA) (Supplementary Table 4).

Interaction network analysis of down-regulated and up-regulated genes with protein–protein interactions was performed using STRING (Search Tool for the Retrieval of Interacting Genes) v.10.0 software (11).

Measurement of plasma progesterone

For measurement of plasma progesterone, peripheral blood was collected by cardiac puncture from *Cd11b*$^{DTR/DTR}$ dams 24 h after administration of DT or PBS on 16.0 dpc. Plasma was obtained after centrifugation at 1300 x g for 10 min at 4°C and progesterone concentrations were measured using an enzyme-linked immunoassay (Mouse/Rat Progesterone Kit, Cat#55-PROMS-E01, ALPCO Diagnostics, Salem, NH, USA), following the manufacturer's instructions.

Cytokine secretion by myometrial and decidual macrophages

Myometrial and decidual tissue was collected from *Cd11b*$^{WT/WT}$ mice on 16.5 dpc, 17.5 dpc and 18.5 dpc, and disaggregated as described above. Single cell suspensions were then incubated with biotin-conjugated rat anti-mouse F4/80 (clone BM8, eBioscience) and streptavidin microbeads (Miltenyi Biotec) for positive selection

using MS columns and a magnetic MACS separator. The purity of isolated macrophages were confirmed to be >90% by flow cytometry to detect F4/80$^+$ cells. Cells were counted using an automatic cell counter (Cellometer Auto 2000; Nexcelom, Lawrence, MA, USA) and plated in a 24-well plate (Fisher Scientific, Waltham, MA, USA) at a density of 1×10^6 cells/mL in complete RPMI medium for culture at 37°C with 5% CO_2. Macrophages were collected 6 h later and the supernatant recovered for analysis of IL-10, IL-12p70, TNF, and TGFβ concentrations using Quantikine ELISA Kits (R&D Systems, Minneapolis, MN, USA).

Cytokine and chemokine concentrations in the maternal circulation and amniotic fluid

C57BL/6 dams were administered LPS or PBS by intra-amniotic injection on 16.5 dpc, with or without adoptive transfer of M2-polarized BMDM, as described above, then 16 h later were anesthetized with avertin (Sigma) for recovery of maternal peripheral blood by cardiac puncture. After euthanasia by cervical dislocation, amniotic fluid was collected from each amniotic sac with a 26G needle. Maternal plasma and amniotic fluid were stored at −20°C until analysis. The ProcartaPlex Mouse Cytokine & Chemokine Panel 1A 36-plex (Invitrogen by Thermo Fisher Scientific, Vienna, Austria) was used to quantify IFNα, IFNɣ, IL-12p70, IL-1β, IL-2, TNF, GM-CSF, IL-18, IL-17A, IL-22, IL-23, IL-27, IL-9, IL-15/IL-15R, IL-13, IL-4, IL-5, IL-6, IL-10, Eotaxin (CCL11), IL-28, IL-3, LIF, IL-1α, IL-31, GRO-α (CXCL1), MIP1α (CCL3), IP10 (CXCL10), MCP-1 (CCL2), MCP-3 (CCL7), MIP-1β (CCL4), MIP-2 (CXCL2), RANTES (CCL5), G-CSF, M-CSF, and ENA-78 (CXCL5) according to the manufacturer's instructions. Plates were read using the Luminex 100 SystemFill (Luminex, Austin, TX, USA), and analyte concentrations were calculated with ProcartaPlex Analyst 1.0 Software (Affymetrix, San Diego, CA, USA). The sensitivities of the assays were: 3.03 pg/mL (IFNα), 0.09 pg/ml (IFNɣ), 0.21 pg/mL (IL-12p70), 0.14 pg/mL (IL-1β), 0.10 pg/mL (IL-2), 0.39 pg/mL (TNF), 0.19 pg/mL (GM-CSF), 9.95 pg/mL (IL-18), 0.08 pg/mL (IL-17A), 0.24 pg/mL (IL-22), 2.21 pg/mL (IL-23), 0.34 pg/mL (IL-27), 0.28 pg/mL (IL-9), 0.42 pg/mL (IL-15/IL-15R), 0.16 pg/mL (IL-13), 0.03 pg/mL (IL-4), 0.32 pg/mL (IL-5), 0.21 pg/mL (IL-6), 0.69 pg/mL (IL-10), 0.01 pg/mL (Eotaxin), 20.31 pg/mL (IL-28), 0.11pg/mL (IL-3), 0.28 pg/mL (LIF), 0.32 pg/mL (IL-1α), 0.45 pg/mL (IL-31), 0.05 pg/mL (GRO-α), 0.13 pg/mL (MIP-1α), 0.26 pg/mL (IP-10), 3.43 pg/mL (MCP-1), 0.15 pg/mL (MCP-3), 1.16 pg/mL (MIP-1β), 0.37 pg/mL (MIP-2), 0.35 pg/mL (RANTES), 0.19 pg/mL (G-CSF), 0.02 pg/mL (M-CSF), and 5.67 pg/mL (ENA-78). Inter-assay and intra-assay coefficients of variation were less than 10%.

Gene expression in the fetal brain and lung upon treatment with M2-polarized macrophages

C57BL/6 dams were administered LPS or PBS by intra-amniotic injection on 16.5 dpc, with or without adoptive transfer of M2-polarized macrophages, as described above, then 16 h later were euthanized to collect the fetal brain and lung. The tissues were stored in RNAlater Stabilization Solution (Invitrogen by Thermo Fisher Scientific, Baltics UAB, Lithuania) until RNA isolation. Total RNA was isolated using QIAshredders, RNase-Free DNase Sets, and RNeasy Mini Kits (QIAGEN), according to the manufacturer's instructions. RNA concentrations and purity were assessed with the NanoDrop 1000 spectrophotometer (Thermo Scientific), and RNA integrity was evaluated with the Bioanalyzer 2100 (Agilent Technologies, Wilmington, DE, USA). Complementary (c)DNA was synthesized using SuperScript III First-Strand Synthesis SuperMix (Invitrogen by Thermo Fisher Scientific, Carlsbad, CA, USA). Gene expression profiling was performed on the BioMark™ System for high-throughput RT-qPCR (Fluidigm, San Francisco, CA, USA) with the TaqMan® gene expression assays (Applied Biosystems, Life Technologies Corporation, Foster City, CA, USA) listed in Supplementary Table 5.

REFERENCES

1. Xu Y, Plazyo O, Romero R, Hassan SS, and Gomez-Lopez N. Isolation of leukocytes from the human maternal-fetal interface. *J Vis Exp.* 2015(99):e52863.
2. Cailhier JF, Partolina M, Vuthoori S, Wu S, Ko K, Watson S, et al. Conditional macrophage ablation demonstrates that resident macrophages initiate acute peritoneal inflammation. *J Immunol.* 2005;174(4):2336-42.
3. Duffield JS, Forbes SJ, Constandinou CM, Clay S, Partolina M, Vuthoori S, et al. Selective depletion of macrophages reveals distinct, opposing roles during liver injury and repair. *J Clin Invest.* 2005;115(1):56-65.
4. Gomez-Lopez N, Romero R, Arenas-Hernandez M, Panaitescu B, Garcia-Flores V, Mial TN, et al. Intra-amniotic administration of lipopolysaccharide induces spontaneous preterm labor and birth in the absence of a body temperature change. *J Maternal-Fetal & Neonatal Med.* 2018;31(4):439-46.
5. Garcia-Flores V, Romero R, Miller D, Xu Y, Done B, Veerapaneni C, et al. Inflammation-induced adverse pregnancy and neonatal outcomes can be improved by the immunomodulatory peptide exendin-4. *Frontiers Immunol.* 2018;9:1291.
6. Faro J, Romero R, Schwenkel G, Garcia-Flores V, Arenas-Hernandez M, Leng Y, et al. Intra-amniotic inflammation induces preterm birth by activating the NLRP3 inflammasome. *Biol Reprod.* 2019;100(5):1290-305.
7. Care AS, Diener KR, Jasper MJ, Brown HM, Ingman WV, and Robertson SA. Macrophages regulate corpus luteum development during embryo implantation in mice. *J Clin Invest.* 2013;123(8):3472-87.
8. Takahashi K, Naito M, Katabuchi H, and Higashi K. Development, differentiation, and maturation of macrophages in the chorionic villi of mouse placenta with special reference to the origin of Hofbauer cells. *J Leukoc Biol.* 1991;50(1):57-68.
9. Adamson SL, Lu Y, Whiteley KJ, Holmyard D, Hemberger M, Pfarrer C, et al. Interactions between trophoblast cells and the maternal and fetal circulation in the mouse placenta. *Dev Biol.* 2002;250(2):358-73.
10. Arenas-Hernandez M, Sanchez-Rodriguez EN, Mial TN, Robertson SA, and Gomez-Lopez N. Isolation of leukocytes from the murine tissues at the maternal-fetal interface. *J Vis Exp.* 2015(99):e52866.
11. Szklarczyk D, Franceschini A, Wyder S, Forslund K, Heller D, Huerta-Cepas J, et al. STRING v10: protein-protein interaction networks, integrated over the tree of life. *Nucleic Acids Res.* 2015;43:D447-52.

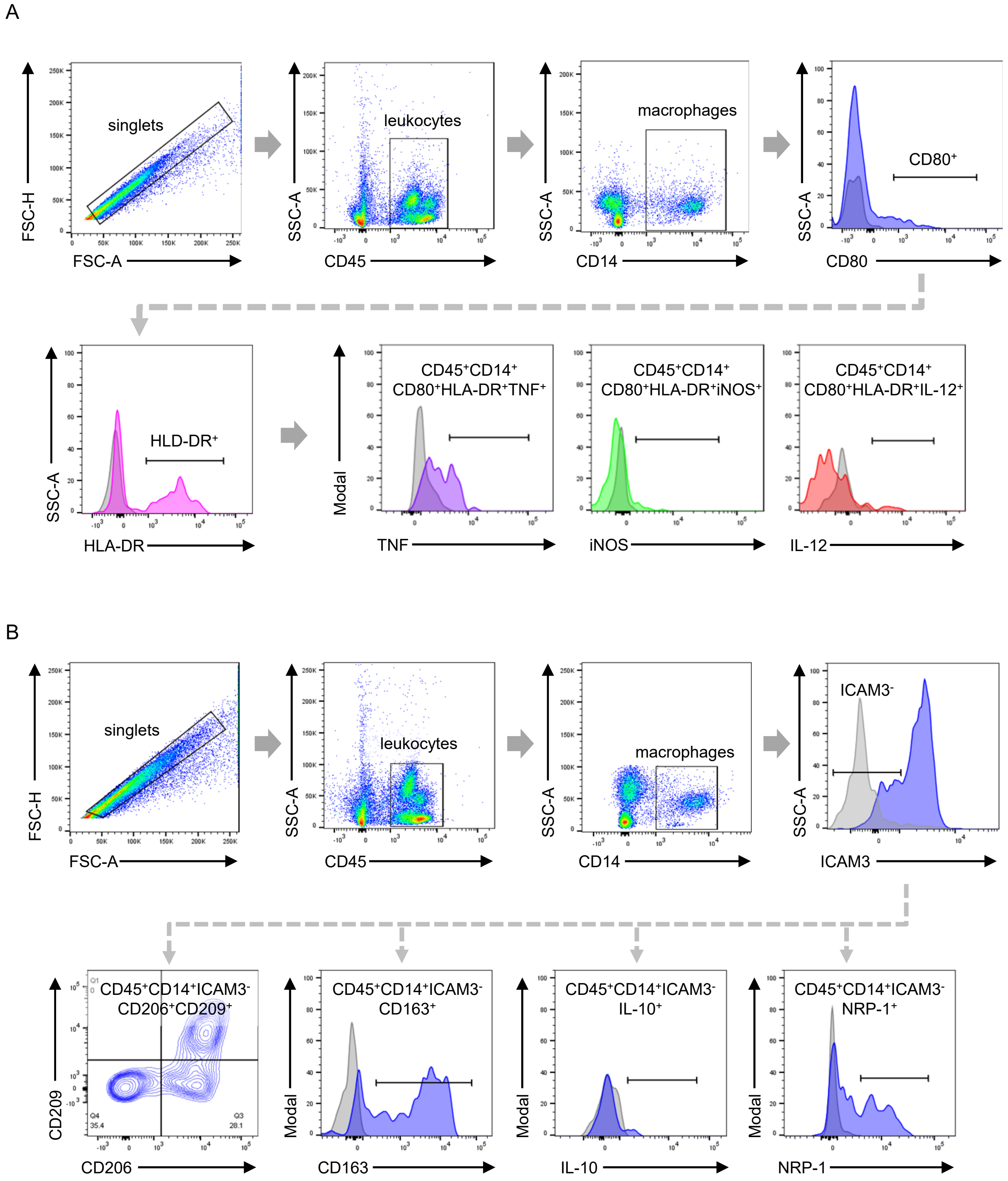

Supplementary Figure 1, related to Figure 1. Gating strategy to identify macrophage subsets at the maternal-fetal interface of women with term or spontaneous preterm birth. (A) Gating strategy used to identify CD45^{+}CD14^{+}CD80^{+}HLA-DR^{+} cells, considered pro-inflammatory macrophages, and subsets expressing TNF, iNOS, or IL12, in uterine decidual tissues. **(B)** Gating strategy used to identify CD45^{+}CD14^{+}ICAM3^{-} cells, considered homeostatic macrophages, and subsets expressing CD206 and CD209, CD163, IL-10, or NRP-1, in uterine decidual tissues.

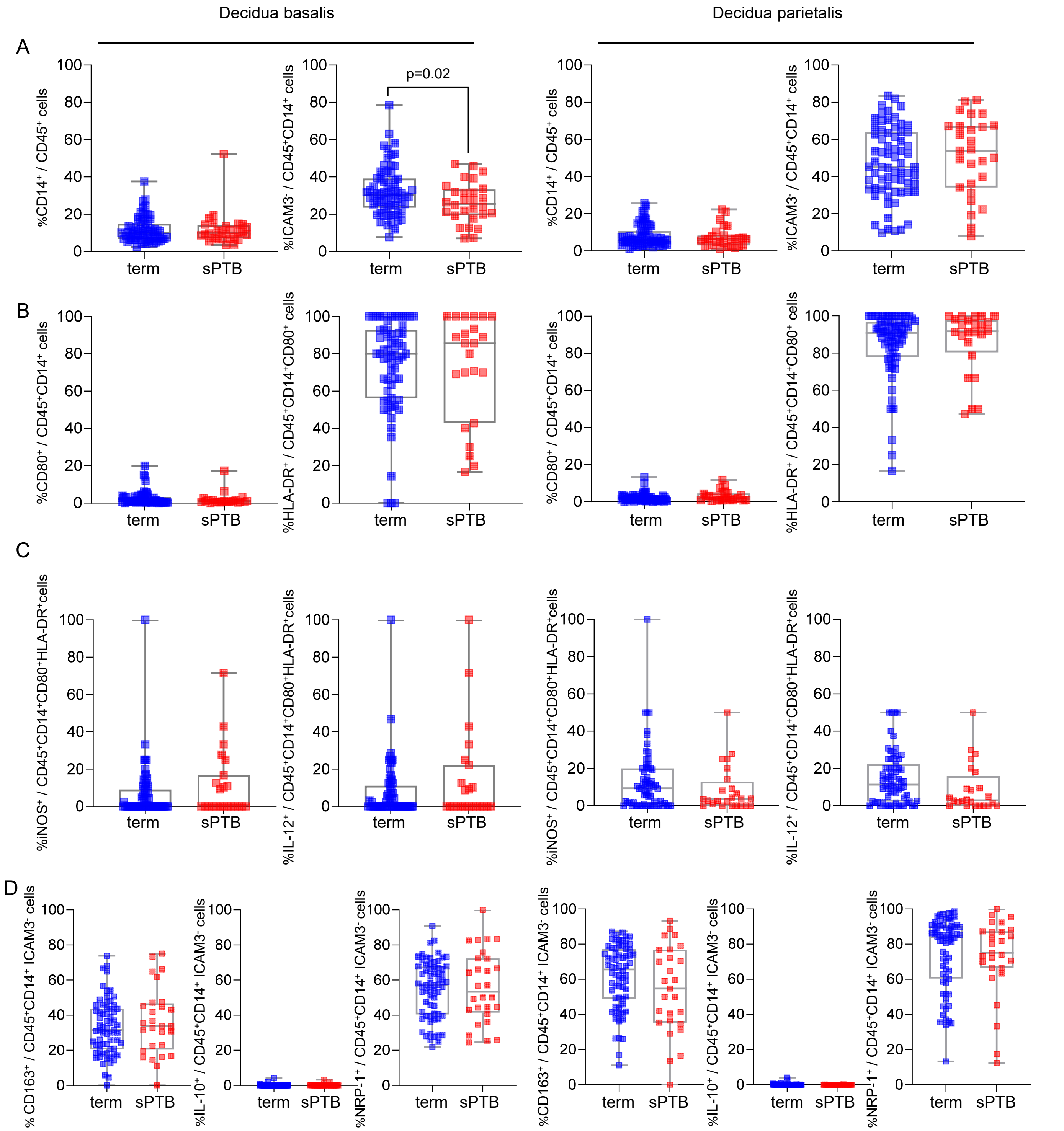

Supplementary Figure 2, related to Figure 1. Macrophage subsets that were not differentially abundant at the maternal-fetal interface of women with spontaneous preterm birth (sPTB), compared to term birth. Frequency of macrophages and macrophage subsets in in the decidua basalis and the decidua parietalis of women who underwent term birth (n = 63-68) or spontaneous preterm birth (n = 23-28). The frequency of **(A)** $CD14^+$ / $CD45^+$ cells and $ICAM3^-$ / $CD45^+CD14^+$ cells; **(B)** $CD80^+$ / $CD45^+CD14^+$ cells and $HLA\text{-}DR^+$ / $CD45^+CD14^+CD80^+$ cells; **(C)** $iNOS^+$ / $CD45^+CD14^+HLA\text{-}DR^+CD80^+$ and $IL\text{-}12^+$ / $CD45^+CD14^+HLA\text{-}DR^+CD80^+$ cells, and **(D)** $CD163^+$ / $CD45^+CD14^+ICAM3^-$, $IL\text{-}10^+$ / $CD45^+CD14^+$ $ICAM3^-$ and $NRP\text{-}1^+$ / $CD45^+CD14^+$ $ICAM3^-$ cells. Data are shown as boxplots where midlines indicate medians, boxes indicate interquartile range, and whiskers indicate minimum/maximum range. Symbols are values from individual women. Data were analyzed by Mann-Whitney U-test. *P* values were considered significant when $P < 0.05$. Demographic and clinical characteristics of the study population are shown in Table 1.

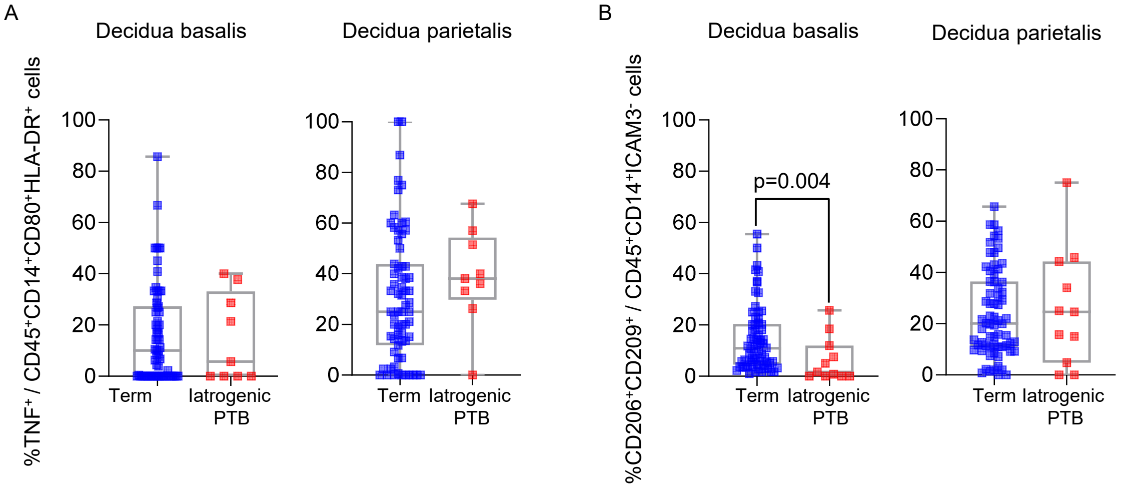

Supplementary Figure 3, related to Figure 1. Macrophage subsets at the maternal-fetal interface of women with iatrogenic preterm birth (PTB), compared to term birth. (A) Frequency of TNF$^+$ / CD45$^+$CD14$^+$CD80$^+$HLA-DR$^+$ cells in the decidua basalis and the decidua parietalis of women who underwent term (n = 63-66) and iatrogenic preterm birth (n = 9-11). **(B)** Frequency of %CD206$^+$CD209$^+$ / CD45$^+$CD14$^+$ICAM3$^-$ cells in the decidua basalis and the decidua parietalis of women who underwent term (n = 67-68) or iatrogenic preterm birth (n = 9-11). Data are shown as boxplots where midlines indicate medians, boxes indicate interquartile range, and whiskers indicate minimum/maximum range. Symbols are values from individual women. Data were analyzed by Mann-Whitney U-tests. *P* values were considered significant when $P < 0.05$.

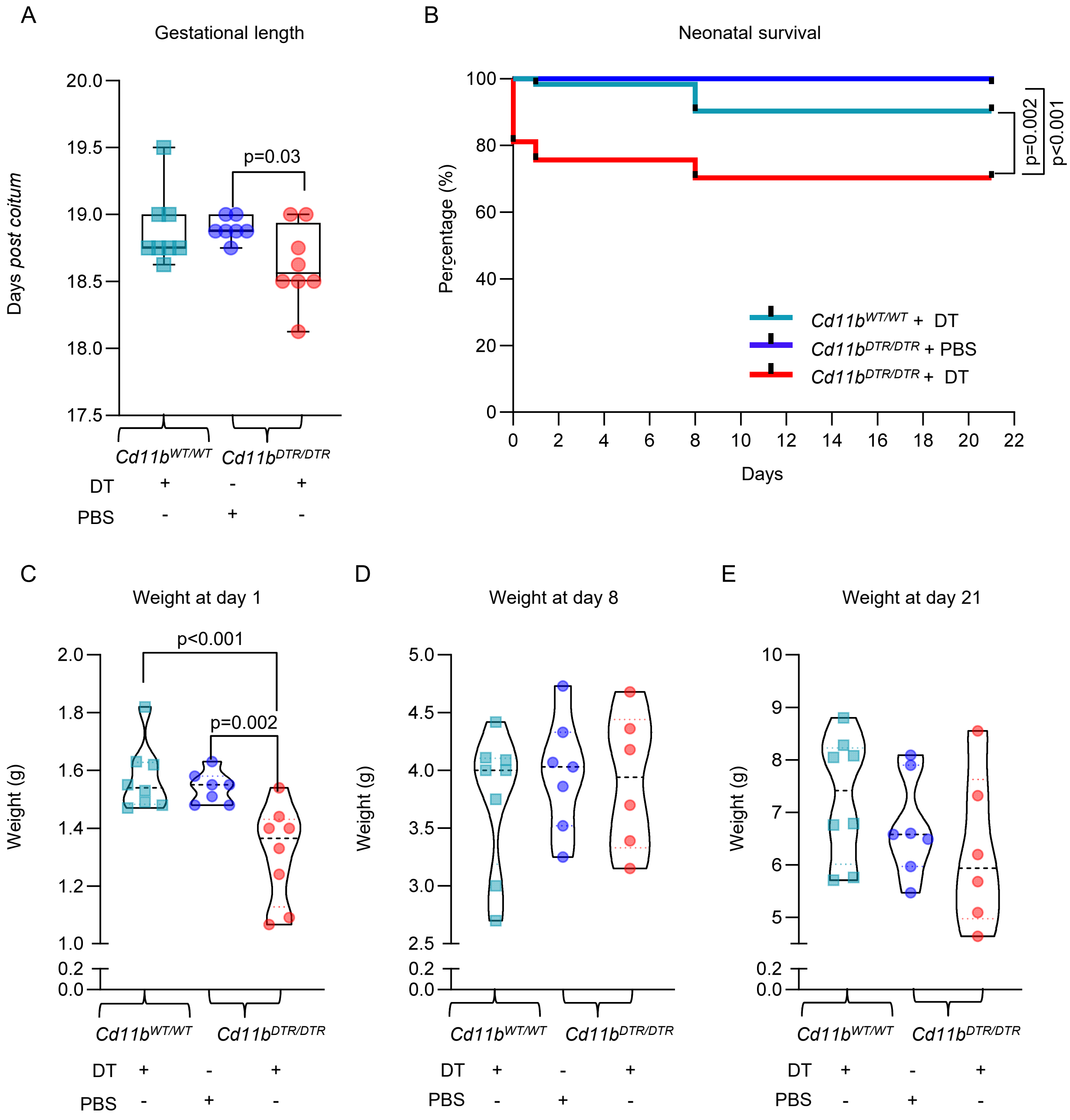

Supplementary Figure 4, related to Figure 2. Depletion of Cd11b⁺ myeloid cells on 17.0 days *post coitum*. *Cd11b*$^{DTR/DTR}$ or *Cd11b*$^{WT/WT}$ dams were administered diphtheria toxin (DT, 25 ng/g, i.p.) or PBS on 16.0 days *post coitum* (dpc). There was no effect of treatment on rate of preterm birth (delivery within 48 h of intervention, eg. ≤19.0 dpc). Parameters shown are **(A)** gestational length presented as boxplots where midlines indicate medians, boxes indicate interquartile range, and whiskers indicate minimum/maximum range medians with interquartile and minimum/maximum ranges, analyzed by Kruskal-Wallis test followed by Mann-Whitney U-test, and **(B)** Kaplan-Meier survival curves showing the percentage survival per litter of neonates at 1, 8, and 21 days postpartum, analyzed by Mantel–Cox tests. **(C, D, E)** Violin plots showing the mean weight per litter of surviving pups at 1, 8, and 21 days postpartum. Symbols are median values from individual dams. Data were analyzed by one-way ANOVA followed by post-hoc t-test. *P* values were considered significant when *P* < 0.05.

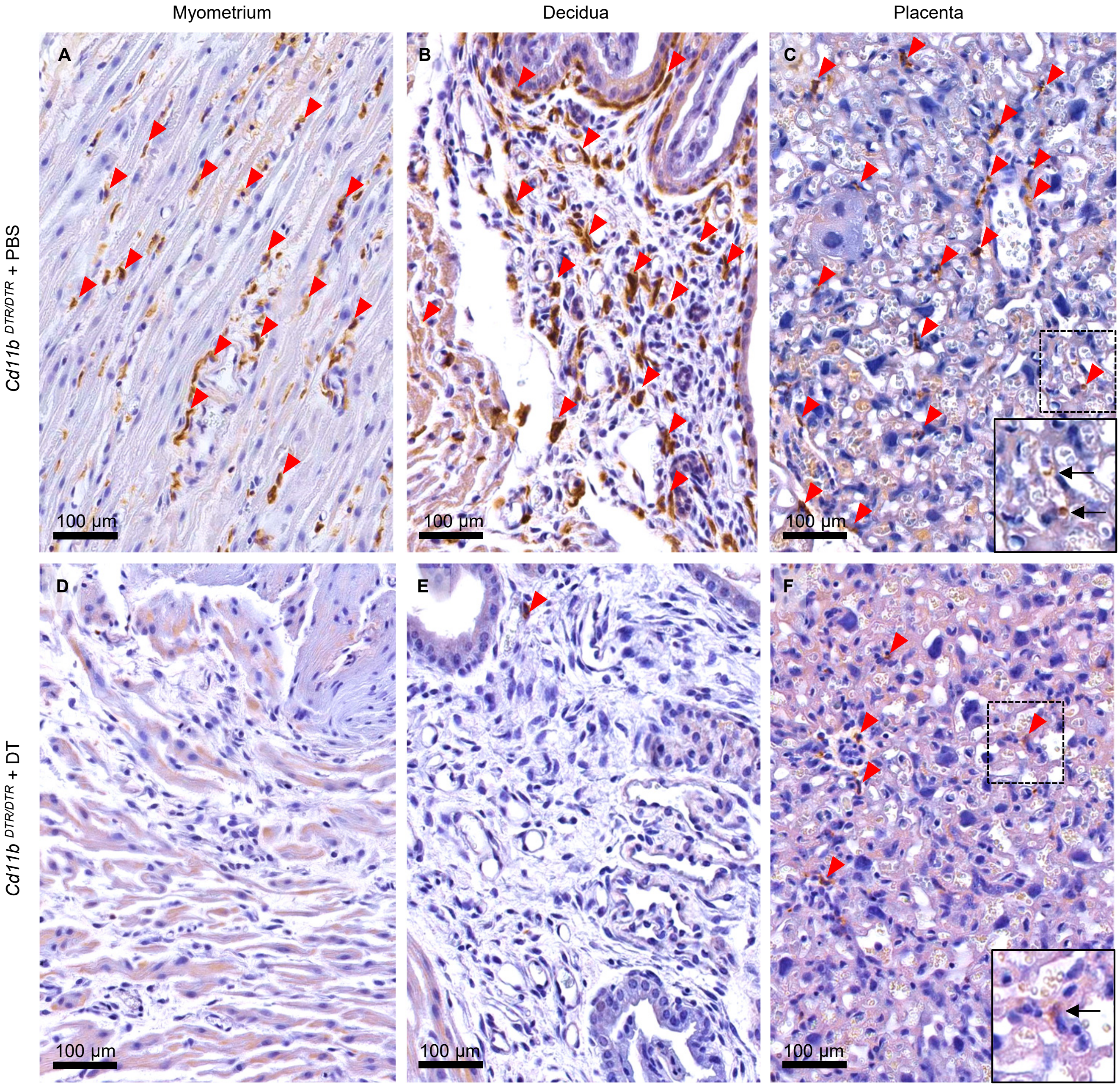

Supplementary Figure 5, related to Figure 3. Administration of diphtheria toxin to *Cd11b*$^{DTR/DTR}$ dams depletes F4/80$^+$ macrophages from the uterus and reduces F4/80$^+$ macrophages in the placenta. *Cd11b*$^{DTR/DTR}$ or *Cd11b*$^{WT/WT}$ dams were administered diphtheria toxin (DT, 25 ng/g, i.p.) or PBS on 16.0 days *post coitum* (dpc). Twenty-four hours post-injection, sections of intact uterine implantation sites (myometrium and decidua) and placenta were collected to detect F4/80$^+$ cells by immunohistochemistry. **(A, B, C)** Myometrium, decidua, and placenta from *Cd11b*$^{DTR/DTR}$ dams administered PBS. **(D, E, F)** Myometrium, endometrium, and placenta from *Cd11b*$^{DTR/DTR}$ dams administered DT. Representative photomicrographs are shown, with the marked area shown at a 2-fold higher magnification in boxes in the lower right hand side of panel C and F. In the placental labyrinth, F4/80$^+$ cells within the intervillous maternal blood space (considered macrophages of maternal origin) can be distinguished from F4/80$^+$ cells within the interstitial villous tissue (considered macrophages of fetal origin, e.g. Hofbauer cells). Macrophages in placental tissue in panel C are located in both the interstitial (fetal) tissue and the maternal blood space, while the macrophages in placental tissue in panel F appear to be located in the interstitial (fetal) tissue. Red arrowheads indicate F4/80$^+$ cells. Black arrows indicate F4/80$^+$ cells within the maternal blood space (panel C) and the fetal interstitial tissue (panel F). Bars are 100 µm.

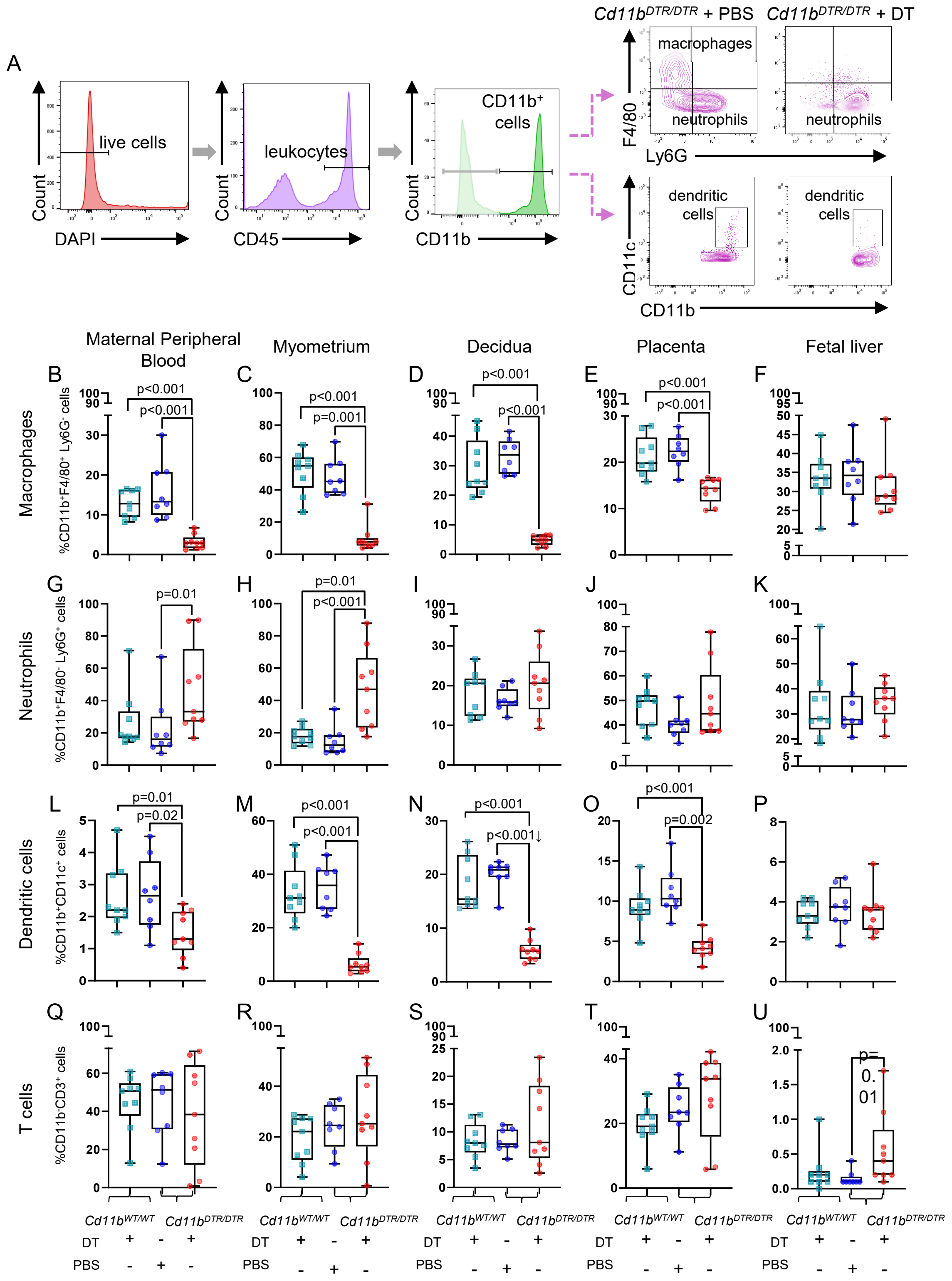

Supplementary Figure 6, related to Figure 3. Effects of depletion of maternal CD11b⁺ myeloid cells on leukocyte populations in maternal and fetal tissues. *Cd11b*$^{DTR/DTR}$ or *Cd11b*$^{WT/WT}$ dams were administered diphtheria toxin (DT, 25 ng/g, i.p.) or PBS on 16.0 days *post coitum* (dpc). Tissues were collected 24 h later for analysis of leukocytes by flow cytometry. **(A)** Gating strategy used to identify macrophages ($CD45^+CD11b^+F4/80^+Ly6G^-$ cells), neutrophils ($CD45^+CD11b^+F4/80^-Ly6G^+$ cells), and dendritic cells ($CD45^+CD11b^+CD11c^+$ cells). (Gating strategy used to identify T cells is shown in Supplementary Figure 8). Proportions of $CD45^+$ leukocytes comprised by macrophages **(B-F)**, neutrophils **(G-K)**, dendritic cells **(L-P)**, and T cells ($CD45^+CD11b^-CD3^+$ cells) **(Q-U),** in the maternal peripheral blood, uterine myometrium, uterine decidua, placenta, and fetal liver (n = 8-9 per group). Data are percentage of $CD45^+$ leukocytes, shown as boxplots where midlines indicate medians, boxes indicate interquartile range, and whiskers indicate minimum/maximum range. Symbols are values from individual dams. Data were analyzed by ANOVA followed by post-hoc t-test. *P* values were considered significant when *P* < 0.05.

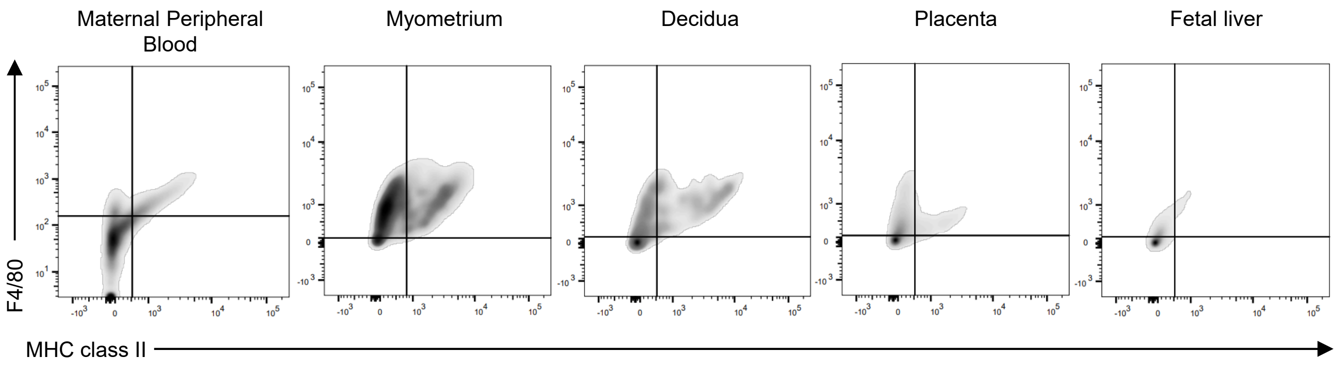

Supplementary Figure 7, related to Figure 3. Expression of MHC class II by F4/80⁺ cells in the maternal peripheral blood, uterine myometrium, uterine decidua, placenta, and fetal liver. Tissues were collected from *CD11b*$^{WT/WT}$ dams on 17.0 days post coitum for analysis of macrophages by flow cytometry using markers F4/80 and MHC class II (see Materials and Methods). Representative density plots are shown.

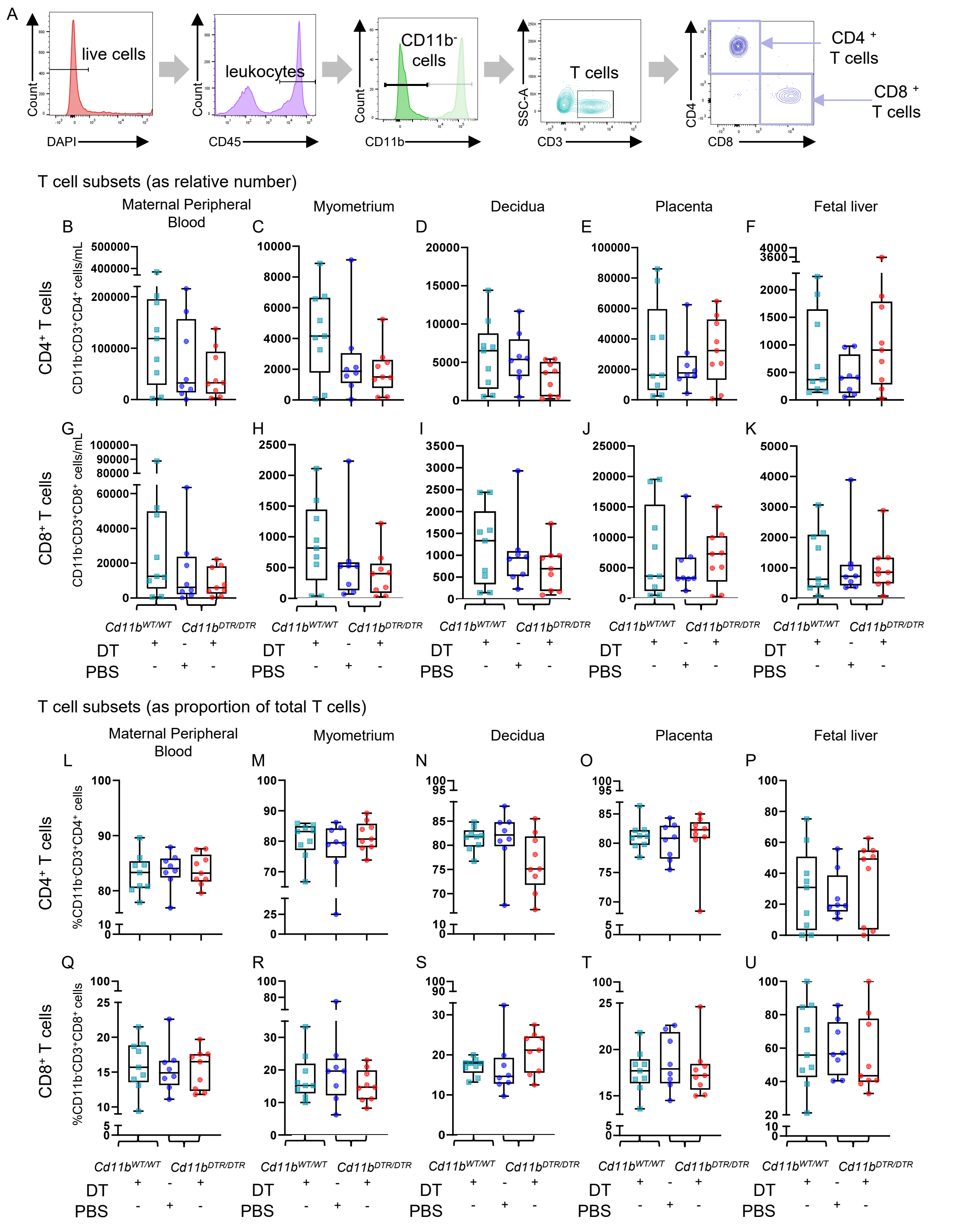

Supplementary Figure 8, related to Figure 3. Effects of depletion of maternal CD11b$^+$ myeloid cells on CD4$^+$ and CD8$^+$ T cell populations in maternal and fetal tissues. (A) Gating strategy used to identify CD4$^+$ T cells (CD45$^+$CD11b$^-$CD3$^+$CD4$^+$ cells), and CD8$^+$ T cells (CD45$^+$CD11b$^-$CD3$^+$CD8$^+$ cells). *Cd11b$^{DTR/DTR}$* or *Cd11b$^{WT/WT}$* dams were administered diphtheria toxin (DT, 25 ng/g, i.p.) or PBS on 16.0 days *post coitum* (dpc). Tissues were collected 24 h later for analysis of T cells by flow cytometry. Numbers of CD4$^+$ T cells (**B-F**) and CD8$^+$ T cells (**G-K**) in the maternal peripheral blood, uterine myometrium, uterine decidua, placenta, and fetal liver (n = 8-9 per group). Proportions of CD4$^+$ T cells (**L-P**), and CD8$^+$ T cells (**Q-U**) in the maternal circulation, myometrium, decidua, placenta, and fetal liver from macrophage-depleted *Cd11b$^{DTR/DTR}$* or control dams (n = 8-9 per group). Data in B-K are number of cells/ ml of digested tissue single cell suspension (see Materials and Methods), and data in L-U are percentage of CD3$^+$ T cells presented as boxplots where midlines indicate medians, boxes indicate interquartile range, and whiskers indicate minimum/maximum range. Symbols are values from individual dams. Data were analyzed by ANOVA followed by post-hoc t-test. *P* values were considered significant when $P < 0.05$.

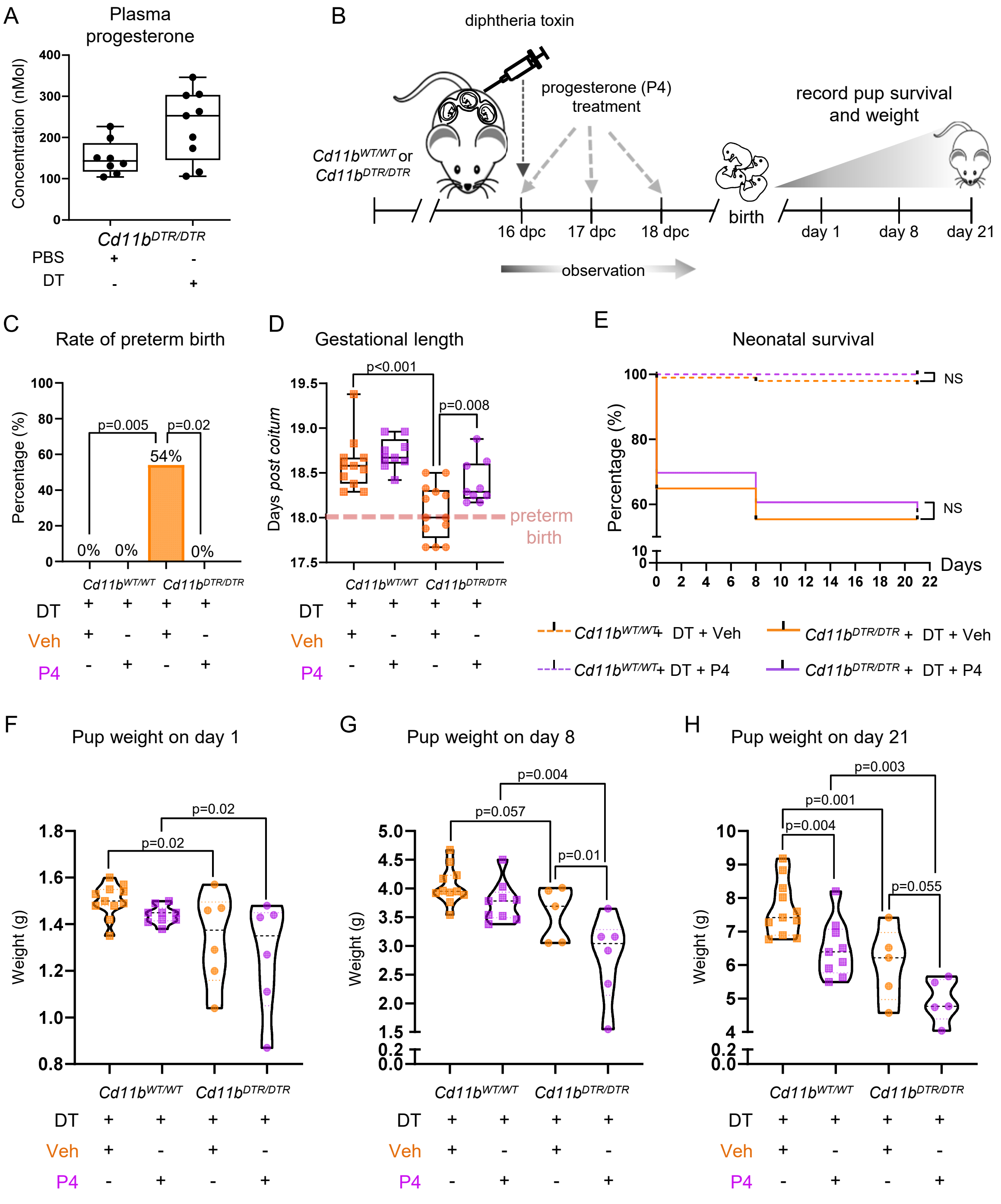

Supplementary Figure 9. Progesterone administration prevents preterm birth but does not reverse adverse neonatal outcomes after depletion of CD11b$^+$ myeloid cells in *Cd11b*$^{DTR/DTR}$ dams. (A) Maternal plasma progesterone concentration in *Cd11b*$^{DTR/DTR}$ dams (mean and SEM, n = 8-9 per group) 24 h after injection of diphtheria toxin (DT, 25 ng/g, i.p.) or PBS on 16.0 dpc. Systemic progesterone concentrations are shown as boxplots where midlines indicate medians, boxes indicate interquartile range, and whiskers indicate minimum/maximum range. Data were analyzed by Mann-Whitney U-test. **(B)** *Cd11b*$^{DTR/DTR}$ or *Cd11b*$^{WT/WT}$ dams were administered diphtheria toxin (DT, 25 ng/g, i.p.) or PBS on 16.0 days *post coitum* (dpc), then administered either progesterone (P4, 200 ng in 100 µl sesame oil, s.c.) or sesame oil (vehicle control; Veh) on 16.0 dpc, 17.0 dpc, and 18.0 dpc. Timing of birth and neonatal outcomes were recorded (all n = 9-13 dams per group). Parameters shown are **(C)** rate of preterm birth (delivery within 48 h of intervention, eg. ≤ 18.0 dpc), analyzed by Fisher's exact test; **(D)** gestational length presented as boxplots where midlines indicate medians, boxes indicate interquartile range, and whiskers indicate minimum/maximum range medians with interquartile and minimum/maximum ranges, analyzed by Kruskal-Wallis test followed by Mann-Whitney U-test. **(E)** Kaplan-Meier survival curves showing the percentage survival per litter of neonates at 1, 8, and 21 days postpartum, analyzed by Mantel–Cox tests. **(F, G, H)** Violin plots showing the mean weight per litter of surviving pups at 1, 8, and 21 days postpartum. Symbols are median values from individual dams. Data were analyzed by one-way ANOVA followed by post-hoc t-test. *P* values were considered significant when $P < 0.05$.

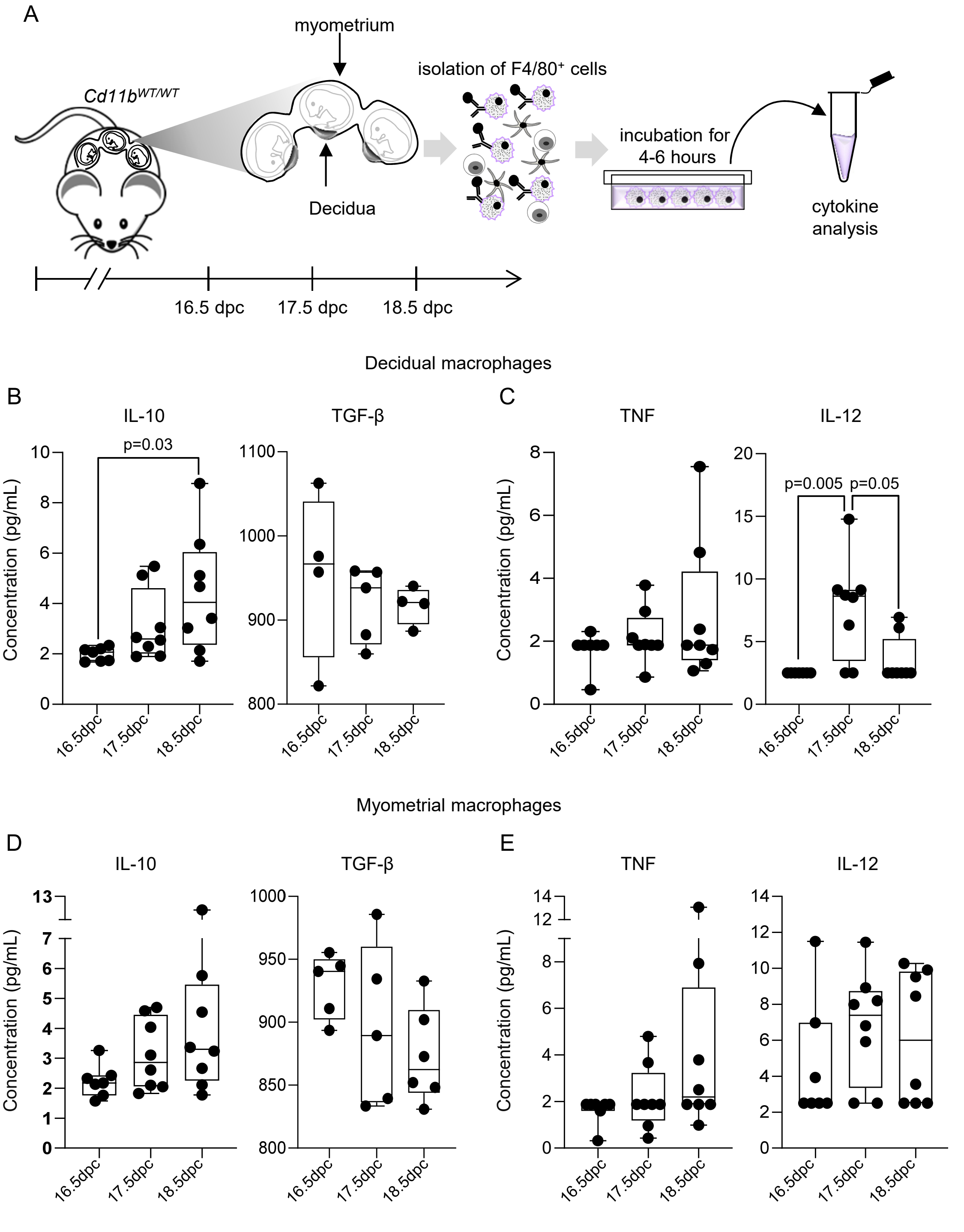

Supplementary Figure 10. Changing dynamics of cytokine synthesis by macrophages in the uterine decidua and myometrium during late gestation in wild-type mice. (A) The myometrium and decidua were collected from *Cd11b*$^{WT/WT}$ dams on 16.5 dpc, 17.5 dpc, or 18.5 dpc. Macrophages were isolated and cultured for 4-6 h, and cytokine concentrations were evaluated in the culture media. **(B, C)** Concentrations of IL-10, TGF-β, TNF, and IL-12 in culture media from decidual macrophages collected on day 16.5 dpc, 17.5 dpc, or 18.5 dpc (n = 7-8 per group). **(D, E)** Concentrations of IL-10, TGF-β, TNF, and IL-12 in culture media from myometrial macrophages collected on day 16.5 dpc, 17.5 dpc, or 18.5 dpc (n = 7-8 per group). Data are presented as boxplots where midlines indicate medians, boxes indicate interquartile range, and whiskers indicate minimum/maximum range. Symbols are median values from individual dams. Data were analyzed by Kruskal-Wallis test followed by Mann-Whitney U-test. *P* values were considered significant when $P < 0.05$.

-8

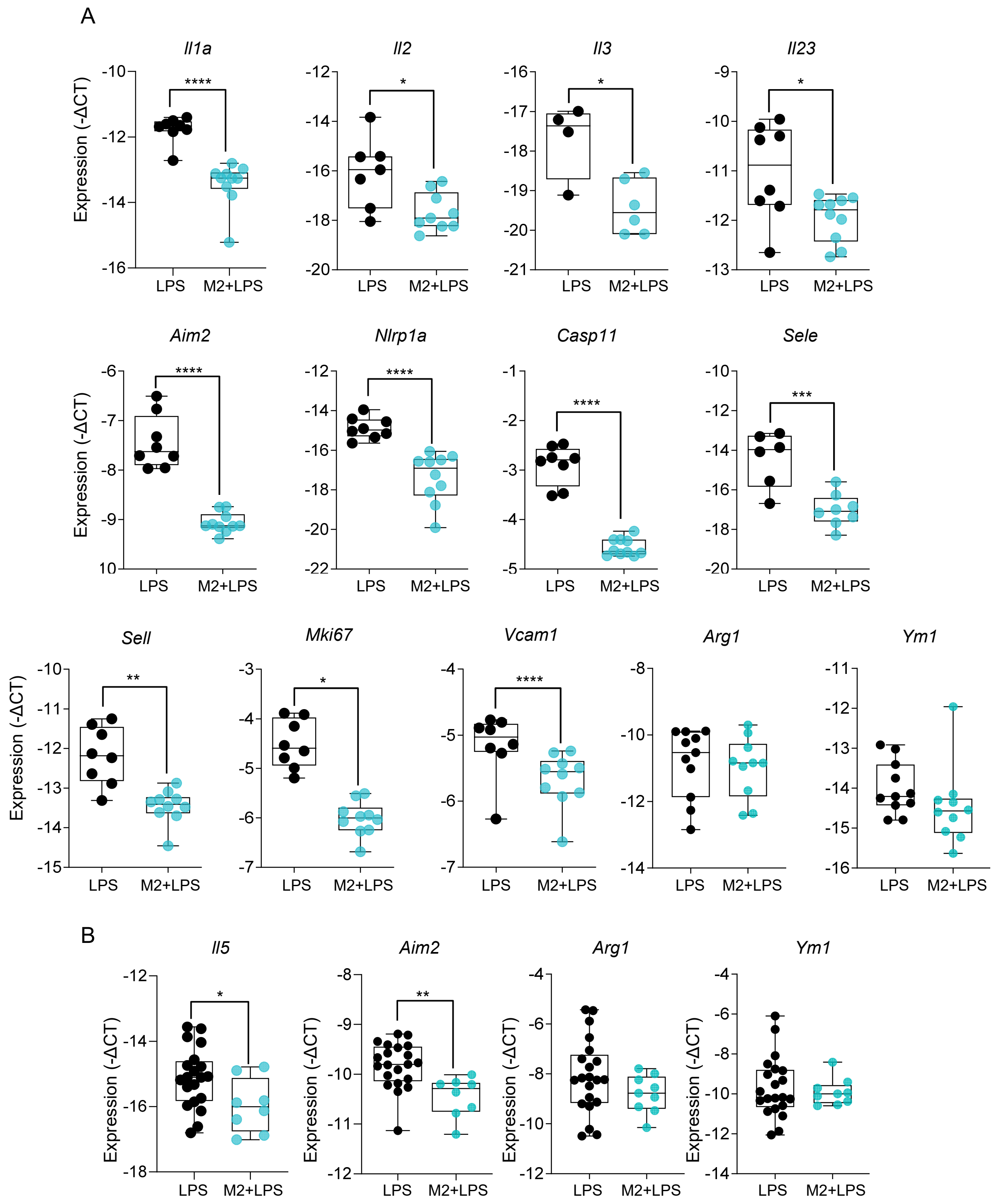

Supplementary Figure 11, related to Figure 8. Effect of adoptive transfer of M2 polarized macrophages to dams on inflammatory gene expression in the brain and lung of fetuses after intra-amniotic administration of LPS. M2-polarized macrophages (M2 Mϕ), or vehicle, were administered intravenously on 15.5 days *post coitum* (dpc) and 16.5 dpc to C57BL/6 dams, followed by intra-amniotic injection with lipopolysaccharide (LPS) on 16.5 dpc (M2+LPS). Control C57BL/6 dams were injected intra-amniotically with LPS only (LPS). Dams were euthanized 16 h after LPS injection to collect fetal brain and lung for evaluation of gene expression. (**A**) Expression of *Il1a, Il2, Il3, Il23, Aim2, Nlrp1a, Casp11, Sele, Sell, Mki67, Vcam, Arg1*, and *Ym1* in the fetal brain (n = 4-10 per group). (**B**) Expression of *Il5, Aim2, Arg1*, and *Ym1* in the fetal lung (n = 8-21 per group). Data are shown as boxplots where midlines indicate medians, boxes indicate interquartile range, and whiskers indicate minimum/maximum range. Symbols are values from individual fetuses. Data were analyzed by Mann-Whitney U-tests. *P* values were considered significant when $P < 0.05$. * $P < 0.05$, ** $P < 0.01$, *** $P < 0.001$, **** $P < 0.0001$.

Supplementary Table 1. Demographic and clinical characteristics of women with iatrogenic preterm birth.

	**Iatrogenic Preterm Birth (n=11)**
Maternal age [years; median (IQR)]	26 (23.5-35.5)
Body mass index [kg/m2; median (IQR)]	28.1 (22.2-35.3)
Gestational age at delivery [weeks; median (IQR)]	33.7 (31.7-36.2)
Birth weight [grams; median (IQR)]	1805 (1501-2220)
Race/ethnicity	
African-American	81.8% (9/11)
White	18.2% (2/11)
Other	0% (0/11)
Primiparity	18.2% (2/11)
Cesarean section	72.7% (8/11)
Indication for Iatrogenic Preterm Birth	
Hypertensive Disorders of Pregnancy	90.9% (10/11)
Preeclampsia	72.7% (8/11)
Chronic Hypertension	9.1 % (1/11)
Eclampsia	9.1 % (1/11)
Placental Abruption	9.1 % (1/11)

[a] One missing datum; IQR, interquartile range

Supplementary Table 2. Antibodies used for human cell analysis.

Antigen	Fluorophore	Clone	Company	Isotype
CD45	V450	HI30	BD Biosciences	Mouse IgG1, κ
CD14	APC-Cy7	MφP9	BD Biosciences	Mouse IgG2b, κ
CD80	PE-Cy7	L307.4	BD Biosciences	Mouse C3H
HLA-DR	PE-Cy5	G46-6	BD Biosciences	Mouse IgG2a, κ
TNF	Alexa Fluor 700	Mab11	BD Biosciences	Mouse IgG1, κ
iNOS	Alexa Fluor 488	4E5	Abcam	Mouse IgG1
IL-12	PE	C11.5	BD Biosciences	Mouse IgG1
ICAM-3	FITC	TU41	BD Biosciences	Mouse IgG1, κ
CD209	PE-Cy7	9E9A8	Biolegend	Mouse IgG2a, κ
CD206	PE-Cy5	19.2	BD Biosciences	Mouse IgG1, κ
CD163	PE-CF594	GHI/61	BD Biosciences	Mouse IgG1, κ
IL-10	PE	JES3-9D7	BD Biosciences	Rat IgG1
NRP1	APC	12C2	Biolegend	Mouse IgG2a, κ

*Antibodies were labelled using the Alexa Fluor 488 Monoclonal Antibody Labeling Kit and Alexa Fluor 680. Antibody Labeling Kit (Life Technologies, Grand Island, NY, USA) following the manufacturer's instructions.

Supplementary Table 3. Antibodies used for mouse cell analysis.

Antigen	Fluorophore	Clone	Company
CD45	APC	30-F11	BD Biosciences
Ly6G	APC Cy7	1A8	BD Biosciences
F4/80	PE	BM8	eBioscience
CD11b	PECy7	M1/70	BD Biosciences
CD8	PE	53-67	BD Biosciences
CD4	APC	RM4-5	BD Biosciences
CD3	FITC	17A2	BD Biosciences
CD11b	PE-CF594	M1/70	BD Horizon
F4/80	APC-eFluor 780	BM8	eBioscience
Egr-2	APC	erongr2	eBioscience
Ym1/2	Phycoerythrin	EPR15263	Abcam
CD3	APC-Cy7	145-2C11	BD Biosciences
CD49b	PE	DX5	BD Biosciences
CD69	PE-Cy7	H1.2F3	BD Biosciences
CD11c	Alexa Fluor 488	N418	eBioscience
CD11c	PECy7	HL3	BD Biosciences
MHC class II	AF700	M5/114.15.2	eBioscience

Supplementary Table 4. Genes assessed using RT² Profiler PCR Array Mouse Innate & Adaptive Immune Response, and RT² Profiler PCR Array Mouse Inflammatory Response & Autoimmunity.

**RT² Profiler PCR Array Mouse Innate & Adaptive Immune Response**			
**UniGene**	**GenBank**	**Symbol**	**Description**
Mm.330510	NM_011318	*Apcs*	Serum amyloid P-component
Mm.19131	NM_009778	*C3*	Complement component 3
Mm.247623	NM_007577	*C5ar1*	Complement component 5a receptor 1
Mm.1051	NM_009807	*Casp1*	Caspase 1
Mm.867	NM_011331	*Ccl12*	Chemokine (C-C motif) ligand 12
Mm.284248	NM_013653	*Ccl5*	Chemokine (C-C motif) ligand 5
Mm.1337	NM_009916	*Ccr4*	Chemokine (C-C motif) receptor 4
Mm.14302	NM_009917	*Ccr5*	Chemokine (C-C motif) receptor 5
Mm.8007	NM_009835	*Ccr6*	Chemokine (C-C motif) receptor 6
Mm.442098	NM_007720	*Ccr8*	Chemokine (C-C motif) receptor 8
Mm.3460	NM_009841	*Cd14*	CD14 antigen
Mm.2209	NM_013488	*Cd4*	CD4 antigen
Mm.271833	NM_011611	*Cd40*	CD40 antigen
Mm.4861	NM_011616	*Cd40lg*	CD40 ligand
Mm.89474	NM_009855	*Cd80*	CD80 antigen
Mm.1452	NM_019388	*Cd86*	CD86 antigen
Mm.1858	NM_001081110	*Cd8a*	CD8 antigen, alpha chain
Mm.28767	NM_007768	*Crp*	C-reactive protein, pentraxin-related
Mm.4922	NM_009969	*Csf2*	Colony stimulating factor 2 (granulocyte-macrophage)
Mm.877	NM_021274	*Cxcl10*	Chemokine (C-X-C motif) ligand 10
Mm.12876	NM_009910	*Cxcr3*	Chemokine (C-X-C motif) receptor 3
Mm.86382	NM_172689	*Ddx58*	DEAD (Asp-Glu-Ala-Asp) box polypeptide 58
Mm.3355	NM_010177	*Fasl*	Fas ligand (TNF superfamily, member 6)
Mm.182291	NM_054039	*Foxp3*	Forkhead box P3
Mm.313866	NM_008091	*Gata3*	GATA binding protein 3

Mm.88795	NM_010391	*H2-Q10*	Histocompatibility 2, Q region locus 10
Mm.439648	NM_010398	*H2-T23*	Histocompatibility 2, T region locus 23
Mm.435508	NM_010493	*Icam1*	Intercellular adhesion molecule 1
Mm.14091	NM_010503	*Ifna2*	Interferon alpha 2
Mm.502	NM_010508	*Ifnar1*	Interferon (alpha and beta) receptor 1
Mm.1245	NM_010510	*Ifnb1*	Interferon beta 1, fibroblast
Mm.240327	NM_008337	*Ifng*	Interferon gamma
Mm.549	NM_010511	*Ifngr1*	Interferon gamma receptor 1
Mm.874	NM_010548	*Il10*	Interleukin 10
Mm.1284	NM_008355	*Il13*	Interleukin 13
Mm.5419	NM_010552	*Il17a*	Interleukin 17A
Mm.1410	NM_008360	*Il18*	Interleukin 18
Mm.15534	NM_010554	*Il1a*	Interleukin 1 alpha
Mm.222830	NM_008361	*Il1b*	Interleukin 1 beta
Mm.896	NM_008362	*Il1r1*	Interleukin 1 receptor, type I
Mm.14190	NM_008366	*Il2*	Interleukin 2
Mm.125482	NM_031252	*Il23a*	Interleukin 23, alpha subunit p19
Mm.276360	NM_021283	*Il4*	Interleukin 4
Mm.4461	NM_010558	*Il5*	Interleukin 5
Mm.1019	NM_031168	*Il6*	Interleukin 6
Mm.38241	NM_008363	*Irak1*	Interleukin-1 receptor-associated kinase 1
Mm.3960	NM_016849	*Irf3*	Interferon regulatory factor 3
Mm.3233	NM_016850	*Irf7*	Interferon regulatory factor 7
Mm.262106	NM_008401	*Itgam*	Integrin alpha M
Mm.275839	NM_008413	*Jak2*	Janus kinase 2
Mm.116844	NM_016923	*Ly96*	Lymphocyte antigen 96
Mm.45436	NM_017372	*Lyz2*	Lysozyme 2
Mm.196581	NM_011949	*Mapk1*	Mitogen-activated protein kinase 1
Mm.21495	NM_016700	*Mapk8*	Mitogen-activated protein kinase 8
Mm.30045	NM_010776	*Mbl2*	Mannose-binding lectin (protein C) 2

Mm.4668	NM_010824	*Mpo*	Myeloperoxidase
Mm.33996	NM_010846	*Mx1*	Myxovirus (influenza virus) resistance 1
Mm.213003	NM_010851	*Myd88*	Myeloid differentiation primary response gene 88
Mm.256765	NM_008689	*Nfkb1*	Nuclear factor of kappa light polypeptide gene enhancer in B-cells 1, p105
Mm.170515	NM_010907	*Nfkbia*	Nuclear factor of kappa light polypeptide gene enhancer in B-cells inhibitor, alpha
Mm.54174	NM_145827	*Nlrp3*	NLR family, pyrin domain containing 3
Mm.28498	NM_172729	*Nod1*	Nucleotide-binding oligomerization domain containing 1
Mm.222633	NM_145857	*Nod2*	Nucleotide-binding oligomerization domain containing 2
Mm.828	NM_009019	*Rag1*	Recombination activating gene 1
Mm.4372	NM_011281	*Rorc*	RAR-related orphan receptor gamma
Mm.2913	NM_013612	*Slc11a1*	Solute carrier family 11 (proton-coupled divalent metal ion transporters), member 1
Mm.277406	NM_009283	*Stat1*	Signal transducer and activator of transcription 1
Mm.249934	NM_011486	*Stat3*	Signal transducer and activator of transcription 3
Mm.1550	NM_011487	*Stat4*	Signal transducer and activator of transcription 4
Mm.121721	NM_009284	*Stat6*	Signal transducer and activator of transcription 6
Mm.477879	NM_019507	*Tbx21*	T-box 21
Mm.203952	NM_174989	*Ticam1*	Toll-like receptor adaptor molecule 1
Mm.273024	NM_030682	*Tlr1*	Toll-like receptor 1
Mm.87596	NM_011905	*Tlr2*	Toll-like receptor 2
Mm.33874	NM_126166	*Tlr3*	Toll-like receptor 3
Mm.38049	NM_021297	*Tlr4*	Toll-like receptor 4
Mm.116894	NM_016928	*Tlr5*	Toll-like receptor 5
Mm.42146	NM_011604	*Tlr6*	Toll-like receptor 6
Mm.23979	NM_133211	*Tlr7*	Toll-like receptor 7
Mm.196676	NM_133212	*Tlr8*	Toll-like receptor 8
Mm.44889	NM_031178	*Tlr9*	Toll-like receptor 9
Mm.1293	NM_013693	*Tnf*	Tumor necrosis factor
Mm.292729	NM_009424	*Traf6*	Tnf receptor-associated factor 6

Mm.20249	NM_018793	*Tyk2*	Tyrosine kinase 2
Mm.328431	NM_007393	*Actb*	Actin, beta
Mm.163	NM_009735	*B2m*	Beta-2 microglobulin
Mm.343110	NM_008084	*Gapdh*	Glyceraldehyde-3-phosphate dehydrogenase
Mm.3317	NM_010368	*Gusb*	Glucuronidase, beta
Mm.2180	NM_008302	*Hsp90ab1*	Heat shock protein 90 alpha (cytosolic), class B member 1

RT² Profiler PCR Array Mouse Inflammatory Response & Autoimmunity

UniGene	GenBank	Symbol	Description
Mm.347398	NM_009744	*Bcl6*	B-cell leukemia/lymphoma 6
Mm.19131	NM_009778	*C3*	Complement component 3
Mm.2408	NM_009779	*C3ar1*	Complement component 3a receptor 1
Mm.477109	NM_009780	*C4b*	Complement component 4B (Childo blood group)
Mm.1283	NM_011329	*Ccl1*	Chemokine (C-C motif) ligand 1
Mm.4686	NM_011330	*Ccl11*	Chemokine (C-C motif) ligand 11
Mm.867	NM_011331	*Ccl12*	Chemokine (C-C motif) ligand 12
Mm.41988	NM_011332	*Ccl17*	Chemokine (C-C motif) ligand 17
Mm.424740	NM_011888	*Ccl19*	Chemokine (C-C motif) ligand 19
Mm.290320	NM_011333	*Ccl2*	Chemokine (C-C motif) ligand 2
Mm.116739	NM_016960	*Ccl20*	Chemokine (C-C motif) ligand 20
Mm.12895	NM_009137	*Ccl22*	Chemokine (C-C motif) ligand 22
Mm.31505	NM_019577	*Ccl24*	Chemokine (C-C motif) ligand 24
Mm.7275	NM_009138	*Ccl25*	Chemokine (C-C motif) ligand 25
Mm.1282	NM_011337	*Ccl3*	Chemokine (C-C motif) ligand 3
Mm.244263	NM_013652	*Ccl4*	Chemokine (C-C motif) ligand 4
Mm.284248	NM_013653	*Ccl5*	Chemokine (C-C motif) ligand 5
Mm.341574	NM_013654	*Ccl7*	Chemokine (C-C motif) ligand 7
Mm.42029	NM_021443	*Ccl8*	Chemokine (C-C motif) ligand 8
Mm.274927	NM_009912	*Ccr1*	Chemokine (C-C motif) receptor 1
Mm.6272	NM_009915	*Ccr2*	Chemokine (C-C motif) receptor 2

Mm.57050	NM_009914	*Ccr3*	Chemokine (C-C motif) receptor 3
Mm.1337	NM_009916	*Ccr4*	Chemokine (C-C motif) receptor 4
Mm.2932	NM_007719	*Ccr7*	Chemokine (C-C motif) receptor 7
Mm.3460	NM_009841	*Cd14*	CD14 antigen
Mm.271833	NM_011611	*Cd40*	CD40 antigen
Mm.4861	NM_011616	*Cd40lg*	CD40 ligand
Mm.439656	NM_009883	*Cebpb*	CCAAT/enhancer binding protein (C/EBP), beta
Mm.28767	NM_007768	*Crp*	C-reactive protein, pentraxin-related
Mm.795	NM_007778	*Csf1*	Colony stimulating factor 1 (macrophage)
Mm.21013	NM_008176	*Cxcl1*	Chemokine (C-X-C motif) ligand 1
Mm.877	NM_021274	*Cxcl10*	Chemokine (C-X-C motif) ligand 10
Mm.131723	NM_019494	*Cxcl11*	Chemokine (C-X-C motif) ligand 11
Mm.4979	NM_009140	*Cxcl2*	Chemokine (C-X-C motif) ligand 2
Mm.244289	NM_203320	*Cxcl3*	Chemokine (C-X-C motif) ligand 3
Mm.4660	NM_009141	*Cxcl5*	Chemokine (C-X-C motif) ligand 5
Mm.766	NM_008599	*Cxcl9*	Chemokine (C-X-C motif) ligand 9
Mm.337035	NM_178241	*Cxcr1*	Chemokine (C-X-C motif) receptor 1
Mm.234466	NM_009909	*Cxcr2*	Chemokine (C-X-C motif) receptor 2
Mm.1401	NM_009911	*Cxcr4*	Chemokine (C-X-C motif) receptor 4
Mm.3355	NM_010177	*Fasl*	Fas ligand (TNF superfamily, member 6)
Mm.246513	NM_010234	*Fos*	FBJ osteosarcoma oncogene
Mm.240327	NM_008337	*Ifng*	Interferon gamma
Mm.874	NM_010548	*Il10*	Interleukin 10
Mm.4154	NM_008349	*Il10rb*	Interleukin 10 receptor, beta
Mm.5419	NM_010552	*Il17a*	Interleukin 17A
Mm.1410	NM_008360	*Il18*	Interleukin 18
Mm.15534	NM_010554	*Il1a*	Interleukin 1 alpha
Mm.222830	NM_008361	*Il1b*	Interleukin 1 beta
Mm.896	NM_008362	*Il1r1*	Interleukin 1 receptor, type I
Mm.253424	NM_008364	*Il1rap*	Interleukin 1 receptor accessory protein

Mm.882	NM_031167	*Il1rn*	Interleukin 1 receptor antagonist
Mm.103585	NM_016971	*Il22*	Interleukin 22
Mm.125482	NM_031252	*Il23a*	Interleukin 23, alpha subunit p19
Mm.221227	NM_144548	*Il23r*	Interleukin 23 receptor
Mm.4461	NM_010558	*Il5*	Interleukin 5
Mm.1019	NM_031168	*Il6*	Interleukin 6
Mm.2856	NM_010559	*Il6ra*	Interleukin 6 receptor, alpha
Mm.3825	NM_008371	*Il7*	Interleukin 7
Mm.3006	NM_008373	*Il9*	Interleukin 9
Mm.1137	NM_008404	*Itgb2*	Integrin beta 2
Mm.2160	NM_023125	*Kng1*	Kininogen 1
Mm.87787	NM_010735	*Lta*	Lymphotoxin A
Mm.1715	NM_008518	*Ltb*	Lymphotoxin B
Mm.116844	NM_016923	*Ly96*	Lymphocyte antigen 96
Mm.213003	NM_010851	*Myd88*	Myeloid differentiation primary response gene 88
Mm.256765	NM_008689	*Nfkb1*	Nuclear factor of kappa light polypeptide gene enhancer in B-cells 1, p105
Mm.2893	NM_010927	*Nos2*	Nitric oxide synthase 2, inducible
Mm.129481	NM_008173	*Nr3c1*	Nuclear receptor subfamily 3, group C, member 1
Mm.292547	NM_011198	*Ptgs2*	Prostaglandin-endoperoxide synthase 2
Mm.112765	NM_138952	*Ripk2*	Receptor (TNFRSF)-interacting serine-threonine kinase 2
Mm.5245	NM_011345	*Sele*	Selectin, endothelial cell
Mm.23987	NM_054096	*Tirap*	Toll-interleukin 1 receptor (TIR) domain-containing adaptor protein
Mm.273024	NM_030682	*Tlr1*	Toll-like receptor 1
Mm.87596	NM_011905	*Tlr2*	Toll-like receptor 2
Mm.33874	NM_126166	*Tlr3*	Toll-like receptor 3
Mm.38049	NM_021297	*Tlr4*	Toll-like receptor 4
Mm.116894	NM_016928	*Tlr5*	Toll-like receptor 5
Mm.42146	NM_011604	*Tlr6*	Toll-like receptor 6
Mm.23979	NM_133211	*Tlr7*	Toll-like receptor 7

Mm.44889	NM_031178	*Tlr9*	Toll-like receptor 9
Mm.1293	NM_013693	*Tnf*	Tumor necrosis factor
Mm.483369	NM_019418	*Tnfsf14*	Tumor necrosis factor (ligand) superfamily, member 14
Mm.103551	NM_023764	*Tollip*	Toll interacting protein
Mm.328431	NM_007393	*Actb*	Actin, beta
Mm.163	NM_009735	*B2m*	Beta-2 microglobulin
Mm.343110	NM_008084	*Gapdh*	Glyceraldehyde-3-phosphate dehydrogenase
Mm.3317	NM_010368	*Gusb*	Glucuronidase, beta
Mm.2180	NM_008302	*Hsp90ab1*	Heat shock protein 90 alpha (cytosolic), class B member 1

Supplementary Table 5. Genes analyzed by TaqMan® gene expression assay.

Gene name	Gene Symbol	Assay ID
Actin, beta	*Actb*	Mm04394036_g1
Glyceraldehyde-3-phosphate dehydrogenase	*Gapdh*	Mm99999915_g1
Glucuronidase, beta	*Gusb*	Mm01197698_m1
Heat shock protein 90 alpha (cytosolic), class B member 1	*Hsp90ab1*	Mm00833431_g1
Absent in melanoma 2	*Aim2*	Mm01295719_m1
Arginase, liver	*Arg1*	Mm00475988_m1
Caspase 1	*Casp1*	Mm00438023_m1
Chemokine (C-C motif) ligand 2	*Ccl2*	Mm00441242_m1
Chemokine (C-C motif) ligand 3	*Ccl3*	Mm00441259_g1
Chemokine (C-C motif) ligand 5	*Ccl5*	Mm01302427_m1
Chemokine (C-C motif) ligand 17	*Ccl17*	Mm01244826_g1
Chemokine (C-C motif) ligand 22	*Ccl22*	Mm00436439_m1
Chitinase-like 3	*Chil3/Ym1*	Mm00657889_mH
Chemokine (C-X-C motif) ligand 1	*Cxcl1*	Mm04207460_m1
Chemokine (C-X-C motif) ligand 9	*Cxcl9*	Mm00434946_m1
Chemokine (C-X-C motif) ligand 10	*Cxcl10*	Mm00445235_m1
CD3 antigen, epsilon polypeptide	*Cd3e*	Mm01179194_m1
Cytotoxic T-lymphocyte-associated protein 4	*Ctla4*	Mm00486849_m1
High mobility group box 1	*Hmgb1*	Mm00849805_gH
Interleukin 1 alpha	*Il1a*	Mm00439620_m1
Interleukin 1 beta	*Il1b*	Mm00434228_m1
Interleukin 2	*Il2*	Mm00434256_m1
Interleukin 3	*Il3*	Mm00439631_m1
Interleukin 4	*Il4*	Mm00445259_m1
Interleukin 5	*Il5*	Mm00439646_m1
Interleukin 6	*Il6*	Mm00446190_m1
Interleukin 9	*Il9*	Mm00434305_m1
Interleukin 10	*Il10*	Mm01288386_m1
Interleukin 12b (IL12p40)	*Il12*	Mm01288989_m1
Interleukin 17a	*Il17a*	Mm00439618_m1
Interleukin 18	*Il18*	Mm00434226_m1
Interleukin 23	*Il23*	Mm00518984_m1
Interleukin 33	*Il33*	Mm00505403_m1
Intercellular adhesion molecule 1	*Icam1*	Mm00516023_m1
Intercellular adhesion molecule 2	*Icam2*	Mm00494862_m1
Interferon gamma	*Ifng*	Mm01168134_m1

Antigen identified by monoclonal antibody Ki 67	*Mki67 (Ki-67)*	Mm01278617_m1
NLR family, CARD domain containing 4	*Nlrc4*	Mm01233151_m1
NLR family, pyrin domain containing 1A	*Nlrp1a*	Mm03047263_m1
NLR family, pyrin domain containing 3	*Nlrp3*	Mm00840904_m1
Nucleotide-binding oligomerization domain containing 1	*Nod1*	Mm00805062_m1
Nucleotide-binding oligomerization domain containing 2	*Nod2*	Mm00467543_m1
Programmed cell death 1	*Pdcd1*	Mm01285676_m1
PYD and CARD domain containing	*Pycard*	Mm00445747_g1
SR-related CTD-associated factor 11	*Scaf11 (Casp11)*	Mm01297328_m1
Selectin; endothelial cell	*Sele (E-selectin)*	Mm00441278_m1
Selectin, lymphocyte	*Sell (L-selectin)*	Mm00441291_m1
Transforming growth factor, beta 1	*Tgfb1*	Mm01178820_m1
Tumor necrosis factor	*Tnf*	Mm00443258_m1
Vascular cell adhesion molecule 1	*Vcam1*	Mm01320970_m1
